# A comprehensive review on heteroatom engineering in fused-ring electron acceptors: from molecular design to device efficiency

**DOI:** 10.1039/d6ra07219d

**Published:** 2026-10-16

**Authors:** Nizamuddin Shaik, Bommaramoni Yadagiri, David Kreher, Surya Prakash Singh, Kamatham Narayanaswamy

**Affiliations:** a Department of Chemistry, SRM University-AP Amaravati Andhra Pradesh-522240 India kamatham.n@srmap.edu.in; b Department of Physical Chemistry and Technology of Polymers, Faculty of Chemistry, Silesian University of Technology M. Strzody Street 9 Gliwice 44-100 Poland; c Centre for Organic and Nanohybrid Electronics (CONE), Silesian University of Technology S. Konarskiego Street 22b Gliwice 44-100 Poland; d Institut Lavoisier de Versailles, UMR CNRS 8180, UVSQ, Université Paris-Saclay Versailles 78035 France; e Polymers and Functional Materials Division, CSIR-Indian Institute of Chemical Technology Uppal Road, Tarnaka Hyderabad-500007 India; f Academy of Scientific and Innovative Research (AcSIR) Ghaziabad Uttar Pradesh-201002 India

## Abstract

Organic solar cells (OSCs) using bulk-heterojunction (BHJ) blends of polymer donors and non-fullerene acceptors (NFAs) have garnered considerable attention in recent years. NFAs, especially fused-ring electron acceptors (FREAs) adopting acceptor–donor–acceptor (A–D–A) and A–DA′D–A structures, have contributed most to high-efficiency OSCs, pushing the power conversion efficiency (PCE) to over 20%. The rapid advancement of novel FREAs is primarily driven by their tunable molecular architectures, broad absorption characteristics, and the ability to finely adjust energy levels and molecular packing through chemical modification. Typically, FREAs consist of coplanar fused aromatic ring systems serving as donor (D) cores, flanked by two highly electron-deficient acceptor (A) end groups. In this review, we summarize recent advances in heteroatom engineering on central fused-ring cores in FREAs for high-efficiency OSCs. The influence of heteroatom incorporation on light absorption, energy levels, electron mobility, and overall photovoltaic performance is systematically discussed. Furthermore, some new guidelines are proposed for future molecular design in the aspect of light absorption, fill factor, driving force, and voltage loss. Finally, we present an outlook on the remaining challenges and highlight promising directions toward the commercialization of OSCs.

## Abstract 2

This review highlights heteroatom engineering of fused-ring electron acceptors for high-efficiency organic solar cells, linking molecular structure to light absorption, energy levels, charge transport, and photovoltaic performance while outlining future design strategies.

## Graphical abstract

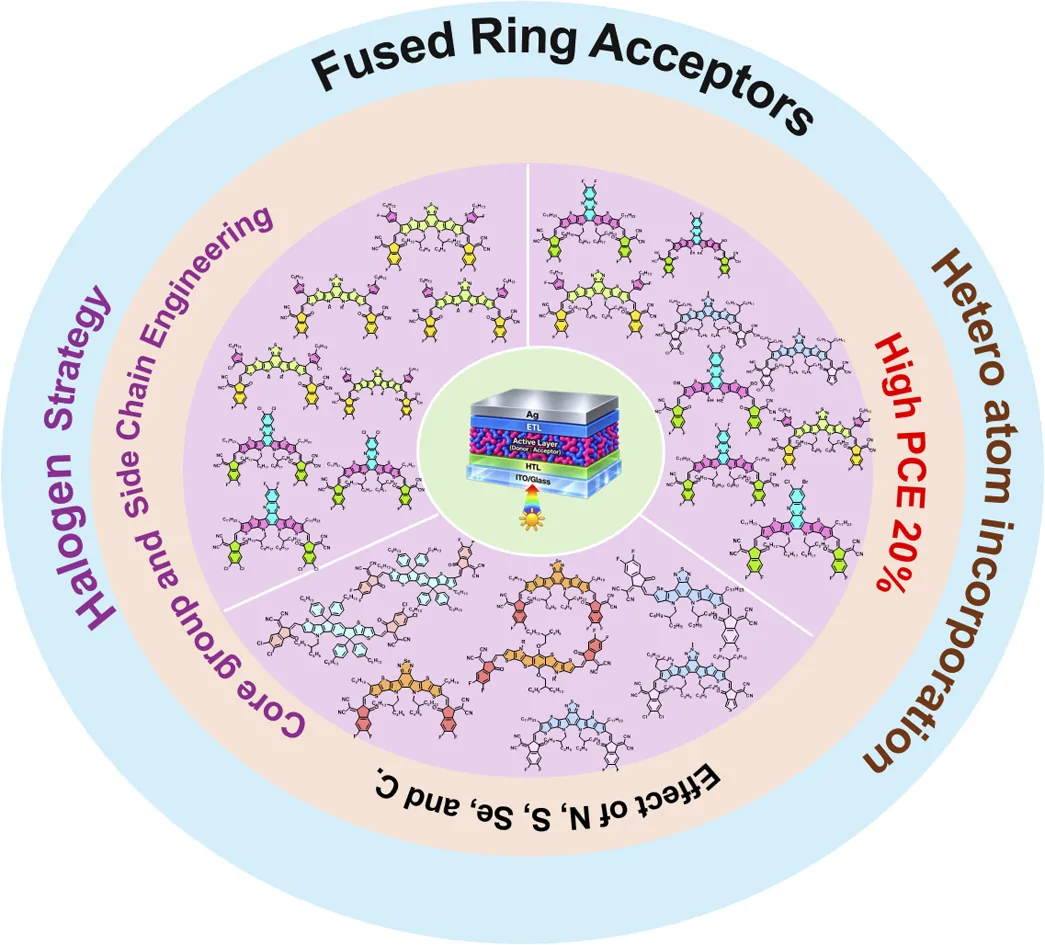

## Introduction

In recent years, the development of environmentally sustainable and renewable energy technologies has emerged as a major scientific strategy for addressing global environmental and energy challenges. Among the various renewable energy resources, photovoltaic technologies, which directly convert solar radiation into electrical energy, have attracted considerable attention owing to their potential to provide clean and sustainable energy. Continued advances in photovoltaic technologies are expected to facilitate the seamless integration of solar harvesting systems into building structures, while simultaneously enabling emerging applications in wearable electronics, portable energy devices, and Internet of Things (IoT) technologies.^1–3^ Among the various next-generation photovoltaic technologies, organic photovoltaics (OPVs) have attracted significant interest owing to their unique advantages, including mechanical flexibility, lightweight, semi-transparency, and compatibility with low-cost, large-area solution-processing and printing techniques, as shown in Fig. 1.^4–11^ The rapid development of OPVs has been largely driven by the emergence of non-fullerene acceptors (NFAs), which possess highly tunable molecular structures that offer broad and intense absorption spectra, favorable energy-level alignment with donor materials, and superior charge-transport properties. In recent years, systematic and rational molecular design strategies have led to the development of numerous high-performance fused-ring electron acceptors (FREAs) exhibiting exceptional optoelectronic and photovoltaic characteristics.^12–19^

**Fig. 1:**
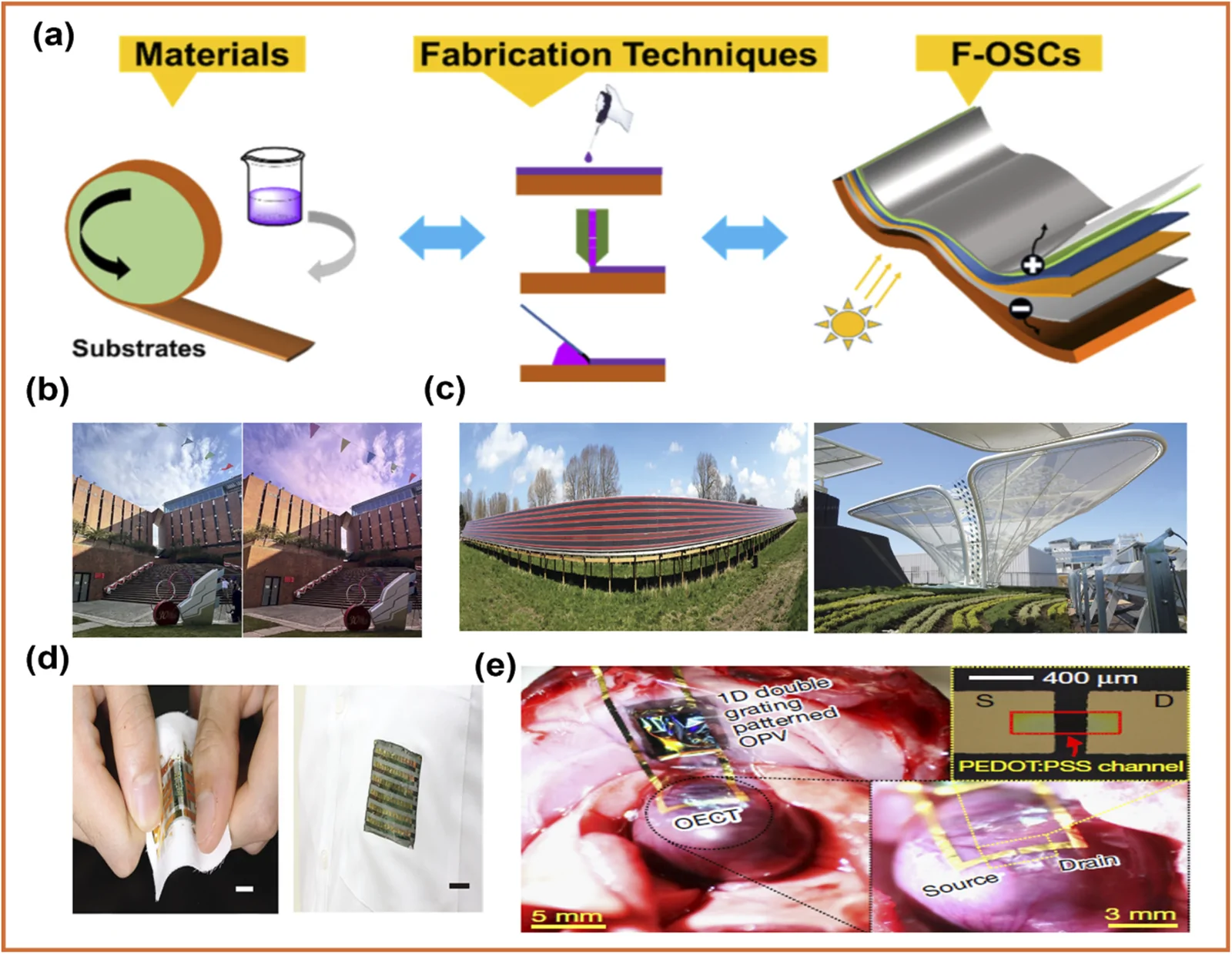
Fabrication methodologies and emerging applications of flexible organic solar cells (F-OSCs). (a) Schematic illustration of representative fabrication techniques employed for the development of F-OSCs, reproduced with permission from ref. 10, copyright 2021, Elsevier; (b and c) building-integrated and semitransparent OSCs, highlighting their potential for sustainable energy harvesting in modern architectural and photovoltaic window applications, reproduced with permission from ref. 7–9, copyright (2025, 2013, 2015) Springer Nature, WILEY-VCH Verlag GmbH & Co. KGaA, Weinheim; (d and e) ultraflexible and implantable OSCs exhibiting excellent mechanical deformability, lightweight characteristics, and compatibility with next-generation wearable and biomedical electronic devices, reproduced with permission from ref. 2 and 10, copyright (2020, 2021) WILEY-VCH Verlag GmbH & Co. KGaA, Weinheim and Elsevier.

A primary objective in FREA's molecular engineering is to achieve red-shifted absorption spectra to maximize solar photon harvesting. For example, Lin *et al.* first reported the A–D–A-type acceptor ITIC, which integrates an electron-rich central donor core flanked by two electron-withdrawing terminal groups. This molecular architecture significantly strengthens intramolecular charge transfer (ICT), thereby extending absorption into the near-infrared (NIR) region and substantially enhancing photocurrent generation.^20^ Building upon this design concept, Yuan and co-workers further advanced NFA development by introducing electron-deficient units into the central fused π-conjugated core, giving rise to A–DA′D–A-type FREAs such as Y6 and its derivatives.^21^ The distinctive crescent-shaped molecular geometry of Y6 promotes highly ordered three-dimensional molecular packing, facilitating efficient charge transport and strong NIR absorption. As a result, Y6-based devices have achieved exceptional short-circuit current (*J*_SC_) values exceeding 25 mA cm^−2^, representing a major milestone in the evolution of high-performance organic solar cells (OSCs).

Despite the remarkable achievement of power conversion efficiencies (PCEs) surpassing 20% in OPVs, substantial energy losses remain a critical factor, an obstacle to further performance improvements, particularly in NIR-absorbing FREA systems.^15,22–27^ Unlike inorganic semiconductors, organic semiconductors typically possess low dielectric constants, resulting in strongly bound excitons with large binding energies that hinder spontaneous charge separation.^28^ Consequently, a substantial driving force is required to dissociate photogenerated excitons into free charge carriers. This limitation can be alleviated through rational molecular design strategies that promote tighter intermolecular packing and enhanced three-dimensional molecular organization. Such structural features facilitate the formation of delocalized intermolecular electronic states, which reduce exciton binding energies and facilitate efficient exciton dissociation.^29–32^ Importantly, these delocalized excited states can bypass conventional charge-transfer (CT) states that are frequently associated with substantial non-radiative recombination losses, thereby enhancing device efficiency. In addition, photovoltaic energy losses are intrinsically linked to the energetic disorder of frontier molecular orbitals, which is largely governed by molecular conformation and intermolecular interactions. Reducing this energetic disorder enables greater separation of the quasi-Fermi levels for electrons and holes, leading to diminished overall energy loss.^33–35^ Therefore, precise control over molecular architecture and molecular packing is essential for the development of next-generation NFAs with minimized energy losses and superior photovoltaic performance.

In this context, heteroatom engineering within the fused core structures of the NFA core has emerged as an effective molecular design strategy for simultaneously modulating electronic structures, intermolecular interactions, and molecular packing.^36–39^ The incorporation of hetero atoms into the central core can finely regulate ICT characteristics between the electron-rich core and electron-deficient terminal groups, often resulting in reduced optical band gaps and red-shifted absorption spectra.^40,41^ In particular, the introduction of chalcogen atoms, such as oxygen, sulfur, and selenium, *etc.*, into the fused-ring backbones can effectively narrow the bandgap through their electron-rich nature, thereby enhancing ICT and extending absorption into longer wavelength regions.^42–45^ Moreover, non-covalent intermolecular interactions can promote molecular packing, exciton delocalization, and charge transport, ultimately contributing to enhanced photovoltaic performance.

Conversely, halogen atoms can be strategically incorporated alongside electron-rich heteroatoms to further regulate molecular electronic structures and intermolecular packing. Owing to their strong electron-withdrawing nature, halogen substituents can effectively tune frontier orbital energy levels while simultaneously promoting molecular packing and other non-covalent interactions. In addition, halogenation can modulate molecular dipole moments and dielectric properties, thereby reducing exciton binding energies and facilitating more efficient charge separation.^46–48^ Collectively, these effects highlight the considerable potential of heteroatom substitution as a versatile molecular engineering strategy for optimizing NFA performance.

Building upon these advances, this review systematically summarizes recent progress in heteroatom engineering within the core moieties of small-molecule NFAs. We focus on the incorporation of different heteroatoms, including nitrogen (N), oxygen (O), sulfur (S), selenium (Se), based on structural modification, into the central fused-ring core unit of FREAs and their influence on photophysical, electrochemical, and photovoltaic properties. A comprehensive analysis of molecular design and structure–property relationships followed by a discussion of the key challenges impeding further efficiency enhancement and large-scale commercialization of OPVs. Finally, prospects on advanced heteroatom engineering strategies are proposed as visible pathways toward next-generation NFAs with minimized energy losses and maximized device efficiency.

## Bulk heterojunction organic solar cells: device architecture, working mechanism, and fundamental principles

Based on the morphology of the photoactive layer, OSCs are commonly categorized into single-layer, bilayer, bulk heterojunction (BHJ), ternary, and tandem architectures, as illustrated in Fig. 2. The first single-layer OPV devices were reported by Kearns and Calvin in 1958, delivering a maximum output power of 3 × 10^−12^ W and a PCE of less than 0.1%. This poor performance was primarily attributed to inefficient exciton dissociation and severe recombination losses of photogenerated charge carriers. Subsequently, Tang and co-workers introduced a bilayer concept in 1986, wherein spatially distinct donor and acceptor layers form a well-defined interface for exciton dissociation. Although this architecture significantly improved charge generation, the overall PCE remained below 1% because of the limited donor–acceptor interfacial area and restricted charge-transport pathways within the individual layers.^49,50^

**Fig. 2:**
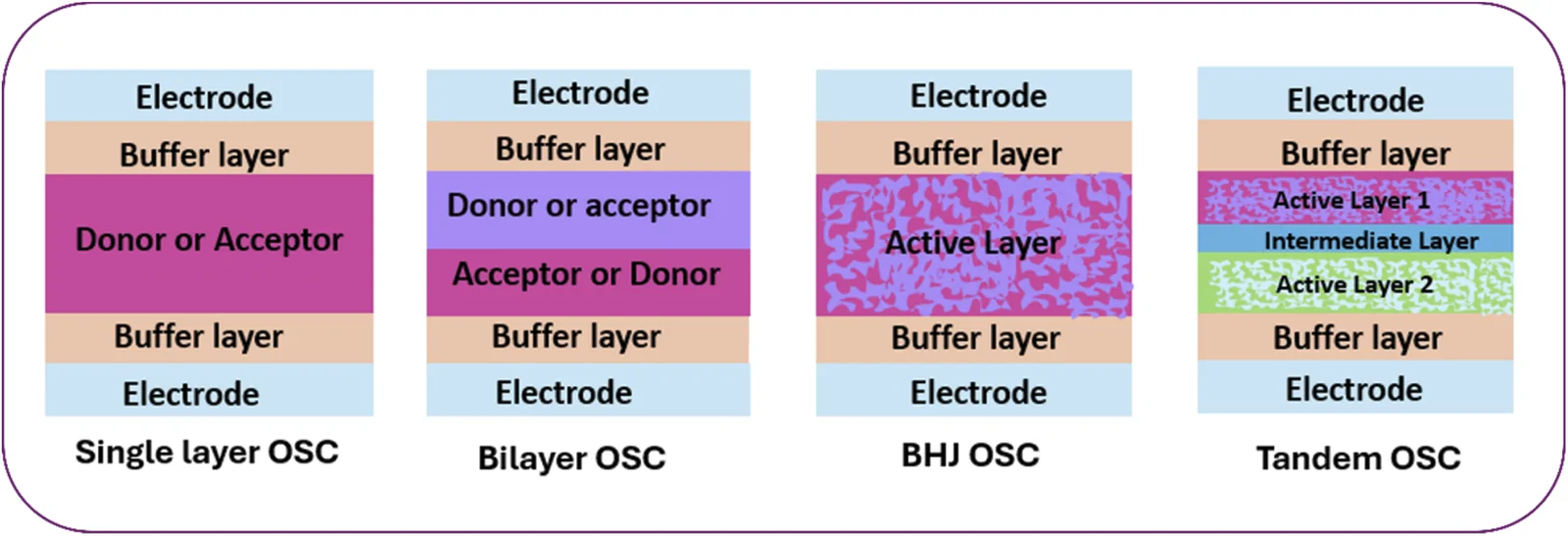
Schematic representation of the OSC device architectures, including single-layer, bilayer, BHJ, and tandem OSC configurations.

A breakthrough occurred in 1995, when Heeger and co-workers developed the BHJ architecture, wherein donor and acceptor materials are intimately blended to form a bicontinuous nanoscale interpenetrating network. This morphology significantly enhances the donor–acceptor interfacial area, thereby facilitating efficient exciton dissociation while simultaneously providing continuous pathways for charge transport. As a result, BHJ architectures have become the dominant platform for high-performance OSCs. Recent advances in donor and acceptor materials, morphology control, and device engineering have enabled BHJ OSCs to achieve PCEs approaching 20%, marking a significant milestone and positioning OSCs as a promising photovoltaic technology.^27,51–53^

Building on the success of BHJ framework, considerable efforts have recently been devoted to the development of ternary and tandem OSCs to further enhance device performance. These advanced architectures exploit the synergistic effects of multiple photoactive materials to achieve broader solar-spectrum utilization, precise energy level tuning, and improved morphological control. In ternary OSCs, the active layer typically adopts either donor : acceptor : acceptor (D : A_1_ : A_2_) or a donor : donor : acceptor (D_1_ : D_2_ : A) configuration. The incorporation of a third component, either an additional donor or acceptor, can improve photovoltaic performance through various mechanisms, such as suppression of energy losses, enhancement of open-circuit voltage (*V*_OC_), and optimization of nanoscale morphology, ultimately leading to improved fill factor (FF) and overall efficiency. Furthermore, Tandem OSCs further extend this concept by vertically stacking two or more photoactive sub-cells connected through an interconnecting layer (ICL). This configuration enables individual sub-cells to harvest complementary regions of the solar spectrum, thereby reducing thermalization losses and maximizing photon utilization. Consequently, tandem OSCs have demonstrated substantially higher PCEs and represent one of the promising directions for next-generation OPV devices.^54–57^

Despite the rapid development of ternary and tandem architectures, BHJ OSCs remain the most extensively investigated and widely adopted device configuration. Their well-established fabrication protocols, tunable nanoscale morphology, and consistently high photovoltaic performance have established BHJ devices as the benchmark platform for both fundamental studies and practical advances in organic photovoltaics.

A typical BHJ OSC comprises a multilayer device architecture, wherein each constituent layer can be independently deposited using an appropriate fabrication technique. The photoactive layer comprises two primary components: an electron donor material, commonly a conjugated polymer, pigment, or oligomer, and an electron acceptor, such as fullerenes or NFA derivatives, or polymeric acceptor materials. This active layer is positioned between an anode and a cathode. To facilitate selective charge extraction and suppress interfacial recombination, interfacial layers such as a hole transport layer (HTL) and an electron transport layer (ETL) are commonly introduced at the anode and cathode interfaces, respectively. In addition to the conventional device structure, an alternative inverted device architecture has been adopted in recent years, in which the transparent bottom electrode functions as the cathode and the top electrode serves as the anode. Inverted OSCs generally exhibit enhanced operational and environmental stability, together with improved photovoltaic performance, making them attractive for practical applications.^58,59^ A schematic comparison of conventional and inverted OSCs device architectures is provided in Fig. 3.

**Fig. 3:**
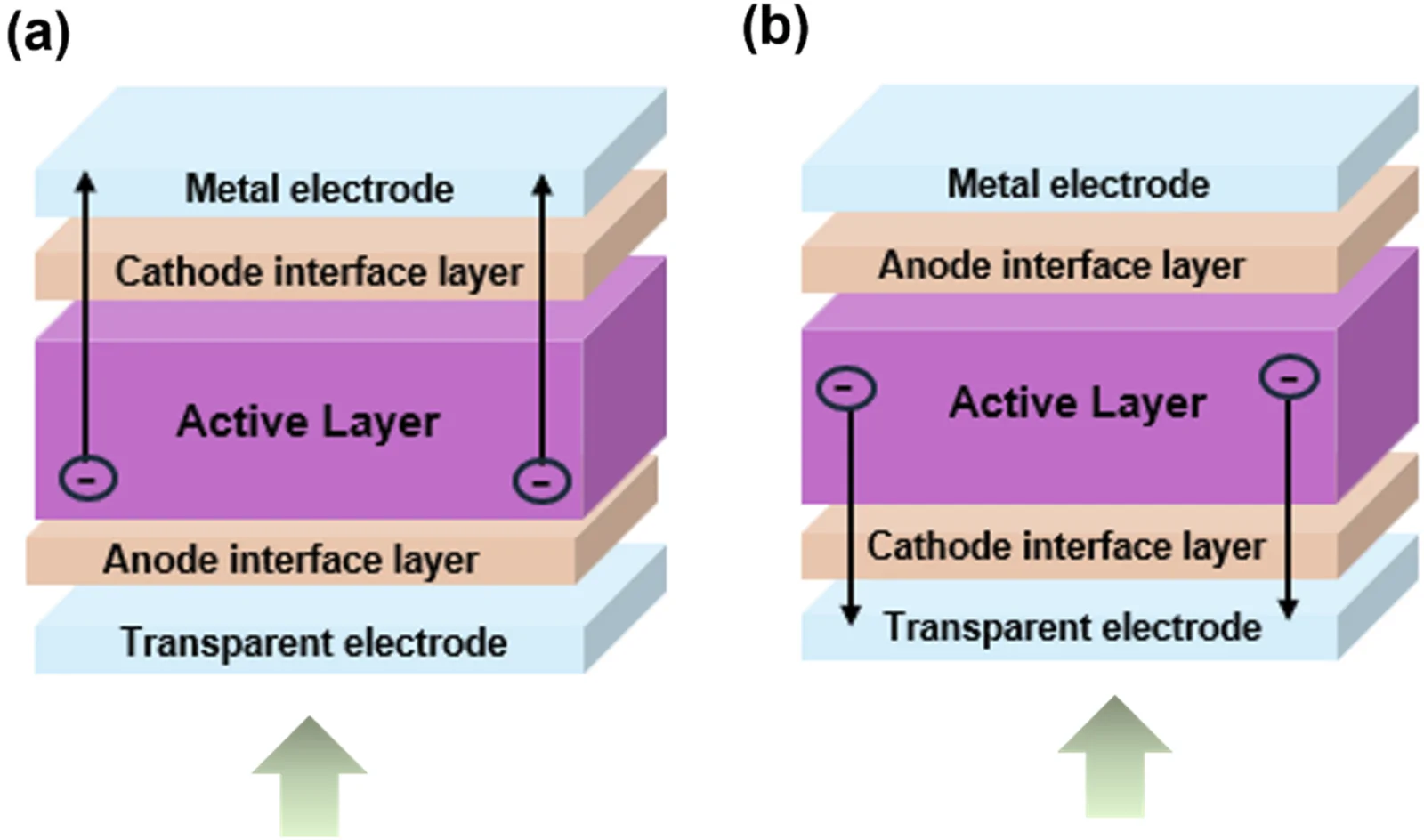
Comparison of (a) conventional and (b) inverted-based OSC device architectures.

## Working principle of BHJ OSCs

The operation of BHJ OSCs involves four fundamental processes: (i) absorption of photons and generation of excitons, (ii) diffusion of excitons to the donor–acceptor (D–A) interface, (iii) exciton dissociation and charge transport, and (iv) charge collection at the electrodes, as shown in Fig. 4a.

**Fig. 4:**
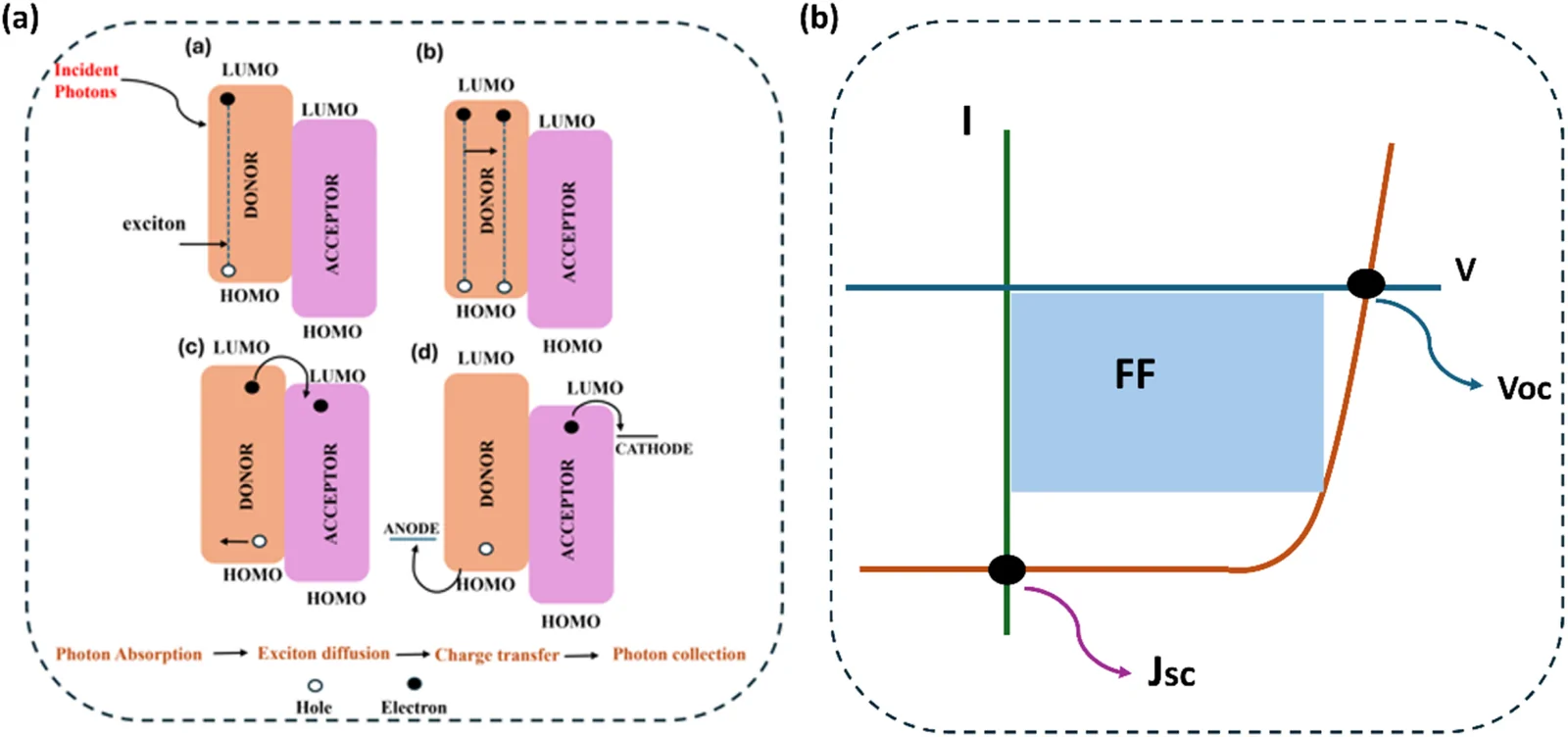
Fundamental operating principles and photovoltaic performance parameters of OSCs. (a) Energy-level diagram illustrating the sequential processes of photon absorption, exciton diffusion, charge transfer, charge separation, and charge collection in a BHJ OSC. (b) Typical current density–voltage (*J*–*V*) curve showing the extraction of key device parameters.

### Light absorption and exciton generation

Efficient light harvesting is essential for achieving high PCE in OSCs. Upon illumination of light from the transparent electrode side, incident photons are absorbed by the photoactive layer, leading to the excitation of electrons from the highest occupied molecular orbital (HOMO) to the lowest unoccupied molecular orbital (LUMO). Similar to inorganic semiconductors, where the electrons are excited from the valence band to the conduction band. Upon photoexcitation, strongly coulombically bound electron–hole pairs, known as excitons, are generated. Owing to the low dielectric constants of organic semiconductors, these excitons typically exhibit binding energies ranging from 0.1 to 1.4 eV.^60^ These excitons must diffuse to the donor–acceptor interface to avoid recombination losses.

### Exciton diffusion and charge dissociation

Following photoexcitation, excitons migrate toward the donor–acceptor interface, where charge separation occurs. The energy offset between the LUMO levels of the donor and acceptor materials provides the driving force required to overcome the coulombic attraction between electrons and holes, thereby facilitating exciton dissociation into free charge carriers. Because excitons in organic semiconductors generally possess short lifetimes, their diffusion lengths are typically limited to less than 20 nm, considerably shorter than the optical absorption depth of ∼100–200 nm. Consequently, efficient charge generation requires that excitons be generated within a distance comparable to their diffusion length (LD) from the donor–acceptor interface. The exciton diffusion length is defined as the average distance an exciton can travel before recombination. Therefore, the nanoscale morphology and thickness of the photoactive layer play crucial roles in maximizing exciton harvesting and charge-generation efficiency.^61^

### Free charge carriers

After exciton dissociation, the resulting free charge carriers must be transported efficiently through the photoactive layer to their respective electrodes to generate photocurrent. In organic semiconductors, charge transport predominantly occurs *via* hopping between localized states. This process is driven by the built-in electric field established by the work-function difference between the anode and cathode. Typically, the combination of a high-work-function anode and a low-work-function cathode generates an internal electric field that promotes charge separation and directional carrier transport. The movement of charge carriers is governed by both drift under the influence of the internal electric field and diffusion driven by carrier concentration gradients. The efficiency of charge transport is strongly dependent on charge-carrier mobility, which influences both carrier extraction and recombination losses. A major challenge in OSC operation is the recombination of free charge carriers before they reach the electrodes. Insufficient charge mobility or unfavorable morphological features can increase carrier accumulation and recombination, thereby reducing photocurrent generation and overall device performance.^62–64^

### Collection of charge carriers

Charge carriers that successfully avoid recombination are eventually collected at their respective electrodes, generating the external photocurrent. Efficient charge collection requires minimal energetic barriers at the interfaces between the active layer and the electrodes. Therefore, the energy levels of the donor and acceptor materials must be appropriately aligned with the electrodes to facilitate selective charge extraction. Ideally, the LUMO level of the acceptor should align favorably with the cathode work function to enable efficient electron extraction, while the HOMO level of the donor should be compatible with the anode work function to facilitate hole collection. Poor energy-level alignment can result in the formation of Schottky barriers, which impede charge extraction and introduce additional energy losses. The charge-collection efficiency is further influenced by factors such as interfacial charge-transfer kinetics, trap-assisted recombination, energetic disorder, and the density of defect states within the active layer. Consequently, optimizing interfacial energetics and minimizing recombination losses are essential for maximizing device performance.^65,66^

The photovoltaic performance of OSCs is commonly evaluated using the PCE, which is defined as:
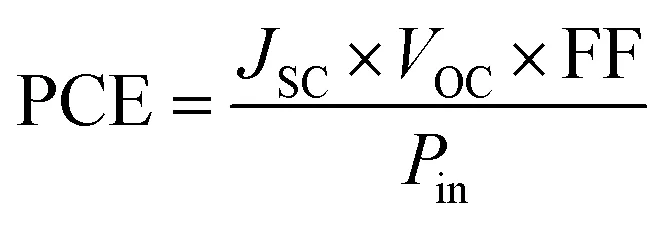


The general parameters of OSCs were represented in Fig. 4b, where *J*_SC_ represents the maximum photocurrent generated by an OSC under short-circuit conditions (*V* = 0). It is governed by several factors, including light absorption, exciton generation and diffusion, charge separation at the donor–acceptor interface, and charge transport and extraction efficiencies. The *V*_OC_ corresponds to the maximum photovoltage obtainable when the external circuit is open (zero current). Although *V*_OC_ is predominantly determined by the energy offset between the HOMO of the donor material and the LUMO of the acceptor material, it is also affected by the energetic position of the CT state, non-radiative recombination losses, interfacial energetics, and active-layer morphology. The FF is a key photovoltaic parameter defined as the ratio of the maximum obtainable electrical power to the product of the *V*_OC_ and *J*_SC_. This parameter reflects the combined effects of charge-carrier transport, recombination kinetics, and extraction efficiency. Consequently, FF is strongly dependent on the molecular packing, phase morphology, charge-carrier mobility, interfacial energetics, and processing conditions of the active layer.^67–69^

### Development history of acceptors for organic solar cells

The creation of novel photoactive semiconducting materials has driven the development of OSCs. According to the timeline, the history of acceptor developments can be classified into three stages: before 1995 (early stage); 1995–2015 (fullerene era); and after 2015 (the non-fullerene era) (Fig. 5). A breakthrough occurred in 1995, with the introduction of PC_61_BM, a fullerene derivative as an efficient electron acceptor material in OSC, marking the beginning of the fullerene era.^51,70^ Then, different fullerene derivatives, including PC_61_BM, PC_71_BM, C_60_ and C_70_ derivatives, such as ICBA, IC_70_BA, ICBM,^71–74^ have been the prominent electron-accepting materials for nearly two decades owing to their excellent electron mobility and favorable charge transport properties. Despite these advantages, fullerene acceptors possess several inherent limitations, such as weak absorption in the visible region and limited molecular energy level tuning. As a result, the PCE of fullerene-based OSCs is restricted to 12%.^75^ To overcome these limitations, extensive efforts were devoted to the development of NFAs. Although early NFAs offered greater structural versatility and tunable optoelectronic properties, their photovoltaic performance initially lagged behind that of fullerene-based systems, with the highest reported PCEs remaining around 6% by the end of 2014.^76,77^

**Fig. 5:**
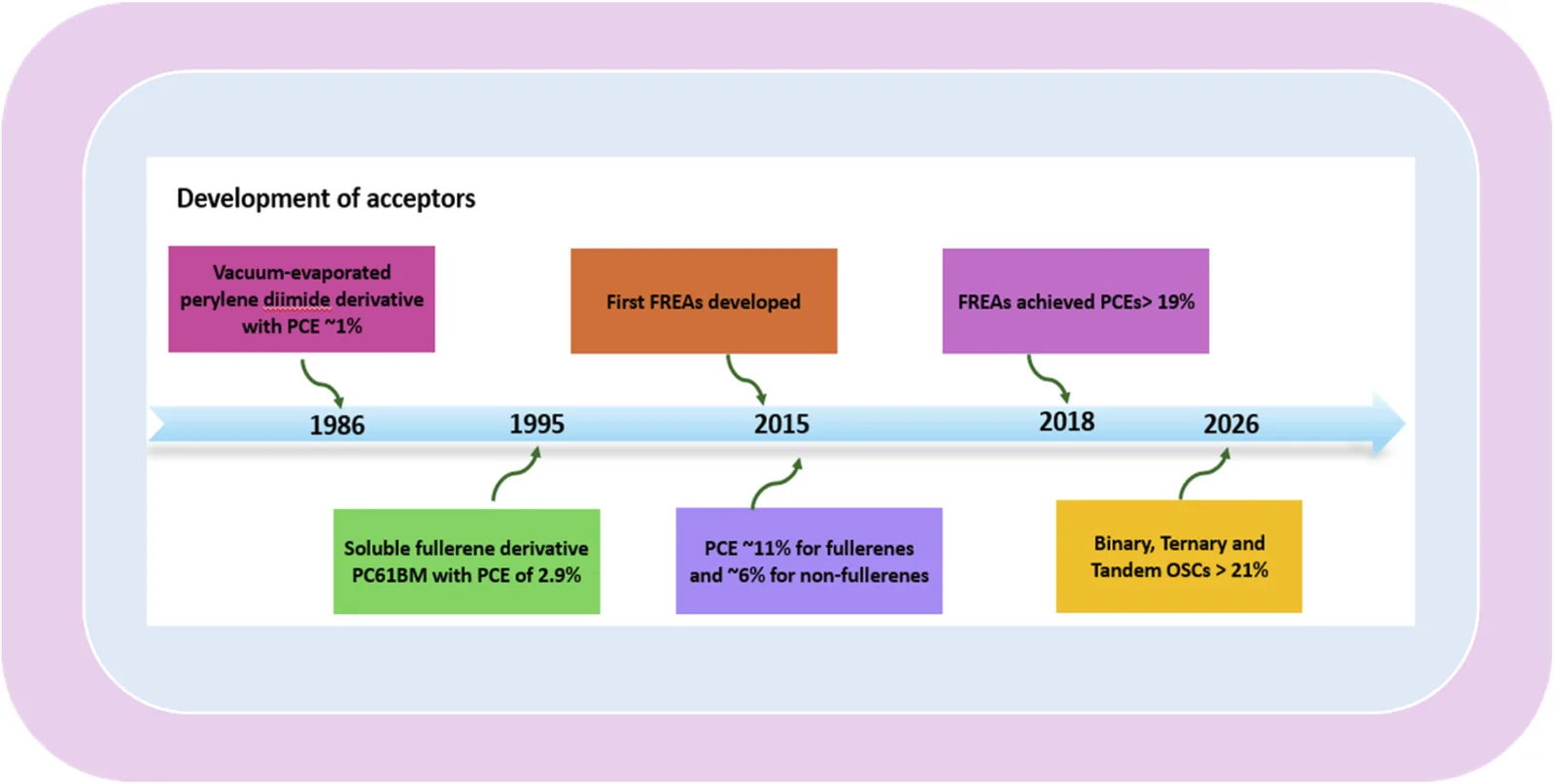
Historical development of electron acceptor materials for OSCs, illustrating major breakthroughs in acceptor design and their impact on device performance.

A transformative advance was achieved in 2014 with the introduction of the A–D–A molecular design strategy. Initially developed for small-molecule donor materials, this design concept was subsequently extended to electron acceptors and rapidly revolutionized NFA development.^78,79^ For example, Zhan and co-workers developed a series of A–D–A-type electron acceptors using indaceno[1,2-*b*:5,6-*b*′]dithiophene as a donor material and 3-(1,1-dicyanomethylene)-1-indanone as an acceptor unit. Among these materials, ITIC represents a milestone and later pioneered the concept of the fused-ring acceptor, also denoted as the FREA.^20,80,81^ Typically, FREAs consist of a fused-ring core with side-chains as the electron-donating unit and electron-withdrawing end groups, which are covalently connected by π-spacer units as shown in Fig. 6. FREAs deliver the distinct benefits over fullerene acceptors:^82^ (i) facile structural engineering and energy levels modulation; (ii) their strong and broad visible to NIR absorption result from strong intramolecular charge transfer benefits light harvesting; (iii) small non-radiative energy losses due to the strong luminescence property; (iv) their rigid and high planar central core unit enable efficient molecular packing and charge transport; (v) high morphological stability ascribed from their non-spherical configuration inhibits migration of molecules. FREAs present a great opportunity in the field of OSCs, promptly boosting PCEs from ∼4% to 20% within 7 years, with newly discovered mechanisms and significant progress towards practical applications.^27,53,83^

**Fig. 6:**
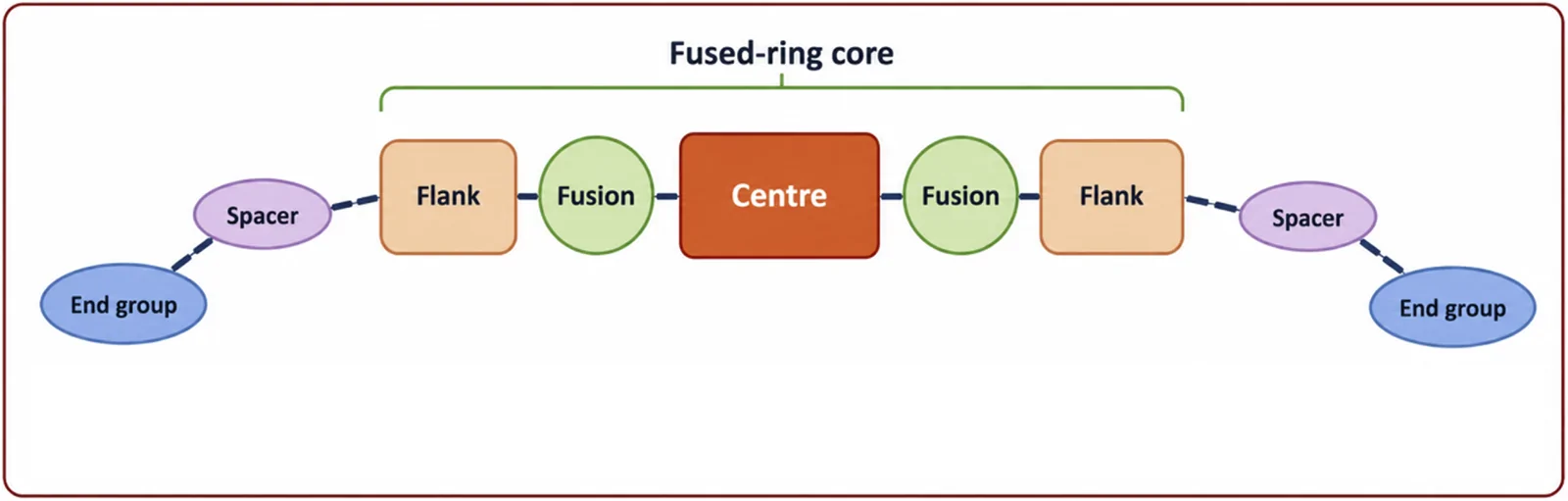
General representative molecular architecture of FREAs.

### Molecular engineering of FREAs for BHJ-OSCs

As shown in Fig. 6, the molecular design of FREAs requires careful optimization of several interdependent parameters that collectively determine photovoltaic performance. For example, elevating the LUMO energy level can increase the *V*_OC_, but may simultaneously reduce the energetic driving force required for efficient exciton dissociation, thereby compromising the *J*_SC_ and FF. Strong and broad absorption in the visible and NIR region results in a high *J*_SC_, but a narrow bandgap (*E*_g_) leads to low *V*_OC_ according to the Shockley–Queisser theory. Furthermore, enhanced molecular crystallinity and electron mobility generally promote efficient charge transport and higher FF, whereas overlarge crystalline domains cause severe charge recombination, resulting in low *V*_OC_, *J*_SC_, and FF. Therefore, achieving high-performance FREAs necessitates a delicate balance between electronic structure, optical properties, and solid-state molecular organization. Precise control over energy levels, absorption characteristics, intermolecular interactions, and molecular packing is essential for minimizing energy losses and maximizing device efficiency.

### ITIC-based FREAs

The introduction of the ITIC NFA in 2015 marked a significant turning point in the advancement of OSCs, effectively reducing the long-standing reliance on fullerene-based acceptors and opening a new direction for photovoltaic research. The emergence of ITIC not only alleviated the long-standing limitations associated with fullerene acceptors but also established a versatile molecular platform for the rational design of next-generation electron acceptors. Compared with conventional fullerene derivatives, NFAs offer several intrinsic advantages, including readily tunable energy levels, strong and broad-spectrum light absorption extending from the visible to the NIR region, enhanced morphological stability, and significantly suppressed non-radiative energy losses. These favorable advantages collectively contributed to remarkable improvements in both the PCE and long-term operational stability of OSC devices.

Among the various NFA materials, the ITIC-based FREAs have played a crucial role in accelerating the performance development of OSCs. Continuous progress in molecular engineering of the ITIC FREA has significantly improved photon harvesting, energy-level alignment, and charge transport processes, thereby accelerating the development of high-efficiency OSCs. One effective strategy involves the incorporation of nitrogen-containing heterocycles into the fused-ring core. For instance, Zhu *et al.* introduced thiophene[3,2-*b*]pyrrole (TP) units into the indacenodithiophene (IDT) backbone, forming a new donor core (PTBTP), and subsequently developed two A–D–A-type acceptors, PTBTP-2F and PTBTP-4F. Owing to nitrogen incorporation, both acceptors exhibited elevated LUMO energy levels, improved film-forming properties, and favorable morphologies. As shown in Fig. 7a, both acceptors exhibited higher absorption coefficients, redshifted absorptions, and lower energy losses relative to the benchmark NFA IT-4F. As a result, when blended with PBDB-T polymer donor, PTBTP-2F-based devices delivered a PCE of 11.14% with a high *V*_OC_ of 0.907 V, whereas PTBTP-4F-based devices reached a superior PCE of 12.33%, accompanied by an enhanced *J*_SC_ of 20.74 mA cm^−2^ and FF of 69.02%.^84^ Luo *et al.* systematically investigated the influence of pyrrole positioning within the IT-4Cl framework through the design of a series of thieno[3,2-*b*]pyrrole-based acceptors such as ThPy1, ThPy2, ThPy3, and ThPy4. When compared to IT-4Cl, the pyrrole-incorporated molecules showed upshifted energy levels, bathochromic absorption, and reduced optical band gaps, as shown in Fig. 7b. More importantly, structural isomerization studies revealed that the positioning of pyrrole units significantly affects molecular planarity, intermolecular interactions, and charge transport behavior. Among these materials, ThPy3 achieved a PCE of 15.3% with a high FF value of 77.1%, while the incorporation of ThPy4 as a third component in PM6:BTP-eC9 blends reached an impressive PCE of 18.43% with a FF of 80.2%.^85^

**Fig. 7:**
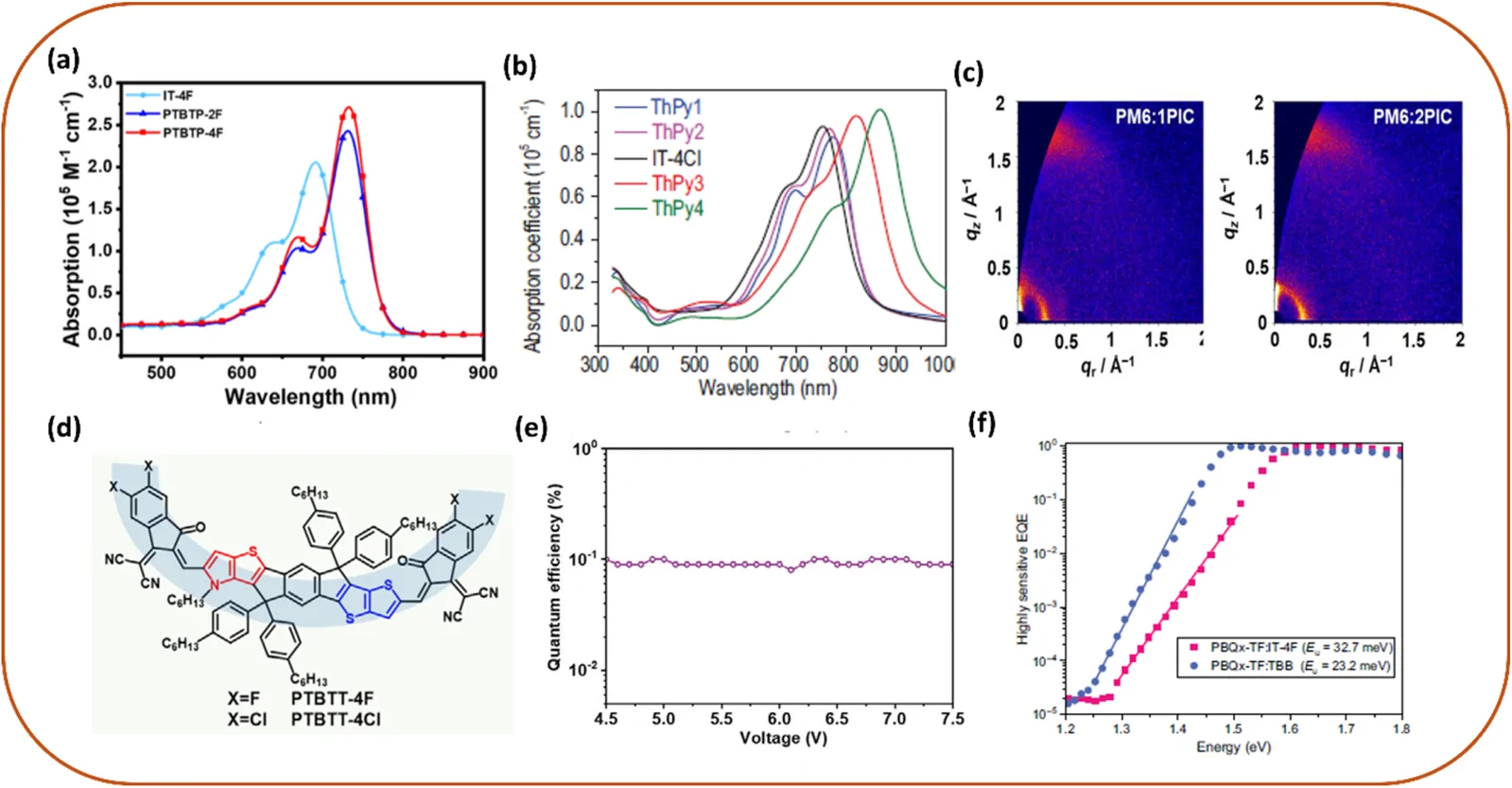
(a) UV-vis absorption spectra of IT-4F, PTBTP-2F, and PTBTP-4F in chloroform solution, reproduced with permission from ref. 84, copyright 2022, Elsevier; (b) thin film absorption spectra of ThPy1, ThPy2, ThPy3, ThPy4 and IT-4Cl, reproduced with permission from ref. 85, copyright 2022, Oxford University Press; (c) two-dimensional GIWAXS pattern of PM6:1PIC and PM6:2PIC blend films, revealing differences in molecular packing and crystallinity, reproduced with permission from ref. 86, copyright 2022, Shanghai, & WILEY-VCH GmbH; (d) molecular structure of the C-shaped fused-ring acceptor illustrating conformational engineering for enhanced optoelectronic properties, reproduced with permission from ref. 87, copyright 2023, Wiley-VCH GmbH; (e) quantum efficiency of the corresponding OLED devices, reproduced with permission from ref. 88, copyright 2024, Wiley-VCH GmbH; (f) high-sensitivity EQE spectra of IT-4F and TBB blends, reproduced with permission from ref. 89, copyright 2024, Chinese Chemical Society.

In addition to positional engineering, expansion of the fused-ring architecture has proven highly effective for enhancing molecular packing and optoelectronic properties. Wang *et al.* proposed a molecular design strategy involving the replacement of a pyrrole unit with a larger pyrrolo[3,2-*b*]pyrrole moiety, leading to the development of two asymmetric NFAs, 1PIC and 2PIC. Compared with 1PIC, the incorporation of the pyrrolo[3,2-*b*]pyrrole unit in 2PIC induced a substantial bathochromic shift of 109 nm, reduced voltage losses, improved electron mobility, elevated frontier molecular orbital energy levels, and tighter molecular packing (Fig. 7c). These synergistic effects facilitated more efficient exciton generation and dissociation, balanced charge transport, and suppressed recombination losses, enabling PM6:2PIC devices to achieve a PCE of 12.6%, substantially higher than the 3.53% obtained for PM6:1PIC devices.^86^

Beyond tuning the electronic structures of the central core, backbone conformational engineering has also emerged as an effective approach for optimizing device performance. Zhu *et al.* developed two asymmetric backbone engineering by incorporating a thiophene[3,2-*b*]pyrrole-based acceptor unit and developed two NFAs, PTBTT-4F/4Cl, featuring a flipped molecular conformation. In contrast to the symmetrical IT-4F and IT-4Cl analogues, the resulting molecular geometry transformed from an “S”-shaped conformation to a more favorable “C”-shaped architecture (Fig. 7d). This conformational modification strengthened intramolecular charge transfer, elevated frontier energy levels, and reduced non-radiative energy losses, thereby contributing to enhanced *V*_OC_ values. Simultaneously, the altered molecular geometry promoted more favorable molecular packing and optimized phase separation, which led to improved *J*_SC_ and FF. Consequently, PTBTT-4F and PTBTT-4Cl devices achieved PCEs of 14.49% and 14.17%, respectively, significantly outperforming their IT-4F and IT-4Cl counterparts.^87^

While the aforementioned studies primarily focused on enhancing charge generation and transport, reducing non-radiative recombination has emerged as another critical design objective. Wang and co-workers developed two medium-bandgap acceptors, FICC-EH and FICC-BO, by introducing different alkyl substituents onto the central fused-ring core. The side-chain engineering strategy effectively enhanced the luminescence properties of both materials, enabling electroluminescence quantum efficiencies approaching 0.1% in OLED devices (Fig. 7e). Benefiting from reduced non-radiative energy losses and improved optoelectronic characteristics, PBQx-TF:FICC-BO devices achieved a PCE of 12.0%, *V*_OC_ of 1.04 V under AM 1.5 G illumination, and an impressive indoor PCE of 25.4% under 1000 lux LED lighting. In contrast, the corresponding FICC-EH-based devices exhibited a lower PCE of 10.4%, highlighting the important role of side-chain engineering in controlling molecular packing and energy-loss pathways.^88^ More recently, suppressing energetic disorder has emerged as a promising strategy for further improving device efficiency. Li and co-workers demonstrated this concept through the development of the ladder-type acceptor TBB. In this design, the sp^3^-hybridized bridging carbon atoms and bulky substituents present in IT-4F were replaced by triisopropylbenzene-substituted pyrrole units, producing a more rigid, planar, and highly conjugated molecular backbone. As a result, TBB exhibited red-shifted absorption and elevated frontier energy levels compared with IT-4F. More importantly, the enhanced backbone rigidity significantly reduced energetic disorder, yielding an Urbach energy of only 23 meV, substantially lower than the 33 meV observed for IT-4F (Fig. 7f). The reduction in energetic disorder facilitated more efficient charge transport, enhanced radiative recombination, and suppressed non-radiative energy losses. Consequently, TBB-based devices achieved a remarkable PCE of 16.2% with a low non-radiative voltage loss of only 0.22 eV, significantly outperforming the corresponding IT-4F-based devices, which exhibited a PCE of 11.5%.^89^

Overall, these studies highlight the pivotal role of nitrogen incorporation in ITIC-based FREAs as a versatile molecular design strategy for optimizing electronic structure, intermolecular interactions, molecular packing, energetic disorder, and non-radiative voltage losses. The synergistic regulation of these factors promotes efficient exciton dissociation, charge transport, and energy-loss suppression, ultimately enabling high-performance organic solar cells. The molecular structures of the representative nitrogen-containing FREAs and their corresponding photovoltaic characteristics are summarized in Fig. 8 and Table 1.

**Fig. 8:**
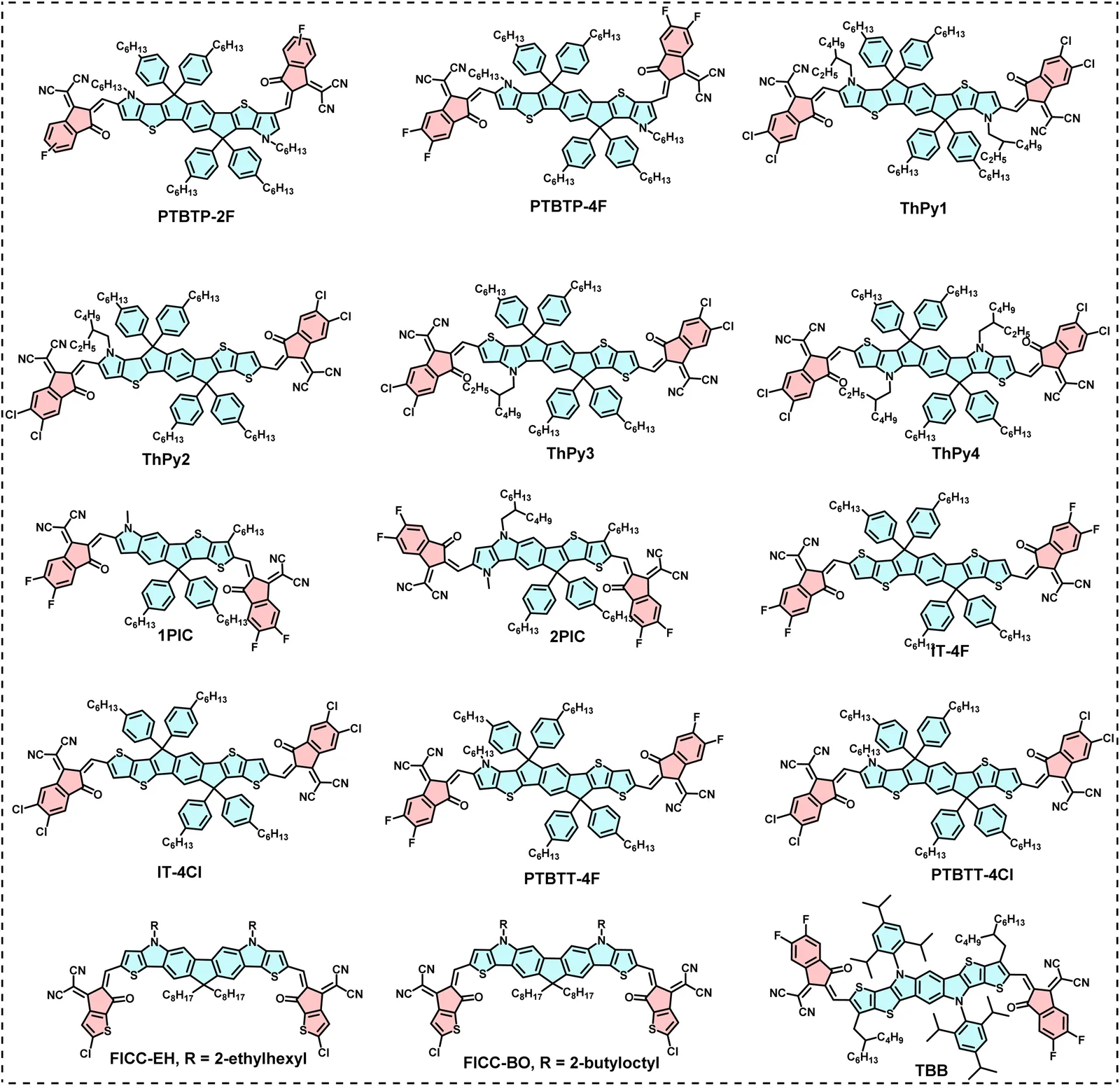
Chemical structures of ITIC-based NFREAs.

**Table 1:** Photovoltaic properties of nitrogen-based ITIC NFAs

Donor	Acceptor	*J*_SC_ (mA cm^−2^)	*V*_OC_ (V)	FF (%)	PCE (%)	*E* ^opt^_g_ (eV)	HOMO (eV)	LUMO (eV)	*E*_loss_ (eV)	Ref.
PBDB-T	IT-4F	19.16	0.722	68.93	9.53	1.55	−5.81	−4.26	0.83	84
	PTBTP-2F	17.89	0.907	68.66	11.14	1.47	−5.56	−4.09	0.62	
	PTBTP-4F	20.74	0.862	69.02	12.33	1.46	−5.57	−4.11	0.66	
PM6	ThPy1	17.68	0.924	71.1	11.6	1.46	−5.59	−3.87	0.61	85
	ThPy2	19.63	0.850	73.8	12.3	1.47	−5.71	−4.01	0.66	
	ThPy3	23.82	0.830	77.1	15.3	1.35	−5.64	−4.04	0.60	
	ThPy4	16.70	0.857	70.1	10.0	1.27	−5.50	−3.95	0.51	
PM6	1PIC	9.56	0.821	43.7	3.53	1.67	−5.69	−3.95	—	86
	2PIC	20.6	0.904	66.1	12.6	1.41	−5.56	−3.89	—	
PBDB-TF	PTBTT-4F	21.43	0.881	76.73	14.49	1.48	−5.68	−4.20	0.634	87
	PTBTT-4Cl	22.81	0.848	73.28	14.17	1.45	−5.70	−4.25	0.640	
	IT-4F	20.80	0.860	73.67	13.18	1.55	−5.76	−4.21	0.739	
	IT-4Cl	22.63	0.768	70.92	12.32	1.48	−5.78	−4.30	0.765	
PBQx-TF	FICC-EH	15.8	1.04	63.6	10.4	1.67	−5.62	−3.78	0.65	88
	FICC-BO	16.8	1.04	68.5	12.0	1.66	−5.66	−3.81	0.66	
PBQx-TF	IT-4F	20.4	0.83	68.0	11.50	1.44	−5.76	−4.00	0.77	89
	TBB	24.3	0.90	74.0	16.20	1.39	−5.56	−3.89	0.59	

### A–DA′D–A type FREAs (Y-series based FREAs)

The emergence of Y-series NFAs has revolutionized the field of OSCs, driving a remarkable improvement in PCE, sharply from ∼13% to >20% within a few years. This rapid development in PCE originated from the unique advantages of Y-series acceptors, which enable simultaneous optimization of light harvesting, energy-level alignment, charge transport, and active-layer morphology. As a result, Y-series NFAs have become the dominant material in OSCs. Although a few review articles have elucidated the structure–property relationships of Y6 molecules,^90^ comprehensive insights into the design principles of the fused-ring core have not yet been fully explained in the literature. In this section, we carefully examine the evolution of Y-series NFAs from the perspective of fused-ring core design. Particular emphasis on how heteroatom substitution, ring fusion, molecular symmetry, and backbone extension regulate the fundamental optoelectronic properties of these materials.

### Molecular design of core structures with heteroatom substitution

Heteroatom substitution has emerged as one of the most powerful molecular engineering strategies for advancing the performance of FREAs. Despite significant progress in OSCs, further enhancement in PCE remains constrained by the intrinsic trade-off between *J*_SC_, *V*_OC_, FF, and substantial energy losses. To address these challenges, the incorporation of heteroatoms (N, S, Se) into the molecular backbone has emerged as an effective molecular engineering strategy. Compared with sp^3^-hybridized carbon, sp^2^-hybridized heteroatoms introduce less steric congestion within the molecular plane, thereby enhancing backbone planarity and facilitating more ordered molecular packing. In particular, the lone-pair electrons of nitrogen atoms can participate in π-electron delocalization, enhancing backbone aromaticity and extending conjugation across the molecular framework. These combined effects strengthen ICT, suppress energetic disorder, and reduce nonradiative energy losses.^91–93^ As a result, heteroatom substitution has been widely employed in the molecular design strategy of both small-molecule acceptors (SMAs) and polymer acceptors (PAs).

Among the various heteroatoms explored, chalcogen atoms have attracted considerable attention owing to their profound influence on the optoelectronic properties of semiconducting materials. Selenium is particularly attractive because of its larger atomic radius, higher polarizability, and more diffuse electron cloud compared with oxygen and sulfur. These characteristics strengthen intermolecular orbital overlap, enhance electronic delocalization, and increase the quinoidal character of conjugated backbones, thereby narrowing the optical bandgap and extending absorption into the NIR region. Moreover, the reduced participation of selenium lone-pair electrons in aromatic π-conjugation facilitates the formation of strong intermolecular Se⋯Se interactions, which promote tighter molecular packing and more efficient charge transport.^93–96^ As a result, selenium incorporation has become a widely adopted strategy for simultaneously enhancing light harvesting, charge-carrier mobility, and photocurrent generation in NIR-absorbing NFAs.

The success of Y-series acceptors has further highlighted the importance of heteroatom engineering within the fused-ring core. A key structural feature underpinning the exceptional performance of these materials is the incorporation of electron-deficient heteroaromatic building blocks into the central fused backbone. Among these, benzo[*c*][1,2,5]thiadiazole (BT) has emerged as one of the most influential motifs because of its strong electron-withdrawing character, rigid planar structure, and ability to promote efficient intramolecular charge transfer when coupled with electron-rich fused-ring segments. The integration of BT into the A–DA′D–A framework represented a breakthrough in NFA design, enabling simultaneous broadband absorption, favorable energy-level alignment, and efficient charge transport. Building upon this foundation, extensive efforts have focused on replacing or modifying the BT unit with structurally related heteroatom-containing analogues, such as benzotriazole (BTA), benzoselenadiazole (BSeD), and phenazine (PZE) (Fig. 9), to further optimize molecular packing, absorption characteristics, energetic disorder, and voltage losses. These developments have established fused-core heteroatom engineering as a key driving force behind the rapid evolution of Y-series NFAs, as discussed in the following sections.

**Fig. 9:**
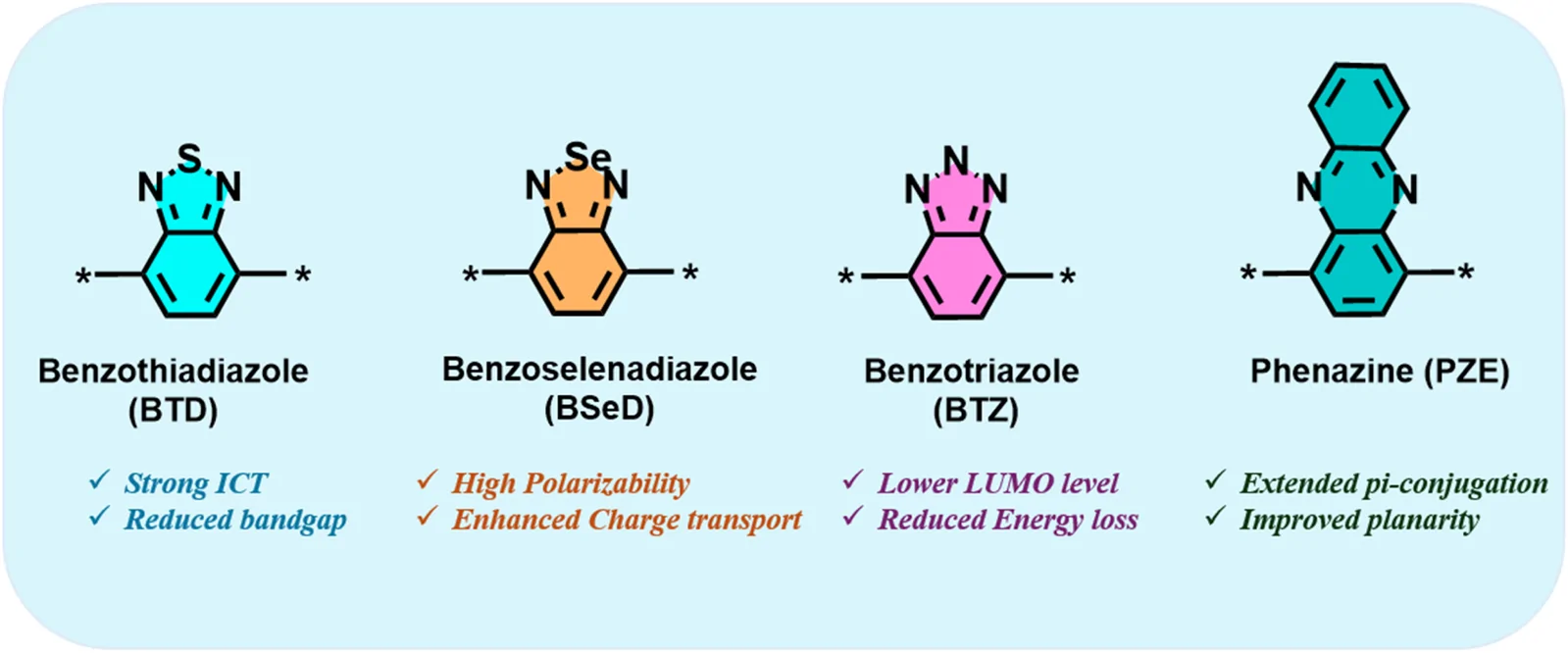
Representative heteroaromatic building blocks, including benzothiadiazole (BTD), benzoselenadiazole (BSeD), benzotriazole (BTZ), and phenazine (PZE), highlighting their key structural and electronic characteristics.

### Benzothiadiazole, benzotriazole, and benzoselenadiazole FREAs

Among the various molecular design strategies developed for FREAs, heteroatom substitution within the central core has emerged as an effective approach for simultaneously tuning the optoelectronic and photovoltaic performance. In particular, nitrogen incorporation in the core moiety has proven to be an effective approach in simultaneously optimizing electronic and morphological properties. For instance, Zou *et al.* reported a novel NFA, BZIC featuring a ladder-type, multifused benzotriazole-based central core (BZTP) end-capped with 1,1-dicyanomethylene-3-indanone (INCN) units. The covalent locking of the thiophene and benzotriazole moieties enhanced π–π interactions, resulting in broad absorption from 600 to 850 nm and a low optical bandgap of 1.45 eV. When blended with the HFQx-T donor, BZIC-based OSCs achieved a PCE of 6.30%, with a *V*_OC_ of 0.84 V, *J*_SC_ of 12.67 mA cm^−2^, and a FF of 0.59.^97^ Liu *et al.* developed a BTA-based acceptor, Y11, by replacing the conventional benzothiadiazole (BT) unit in the Y6-series acceptor. This structural modification yielded a nearly planar molecular confirmation with negligible torsional distortion, leading to a substantial reduction in energetic disorder. Consequently, OSCs based on a PM6:Y11 active layer achieved a high PCE of 16.54% while exhibiting an exceptionally low non-radiative recombination loss of 0.17 eV.^34^ Further advancing this approach, Zhu *et al.* designed an A–DA′D–A type acceptor, Y18, featuring a BTA-fused electron-deficient core. As shown in Fig. 10a, compared with Y6, Y18 demonstrated broadened absorption, an elevated LUMO energy level, and a lower energy loss of 0.53 eV, which collectively increased *V*_OC_ and FF, enabling the PM6:Y18 device to achieve a PCE of 16.52%, further boosted to 17.11% through a ternary blend approach.^98^

**Fig. 10:**
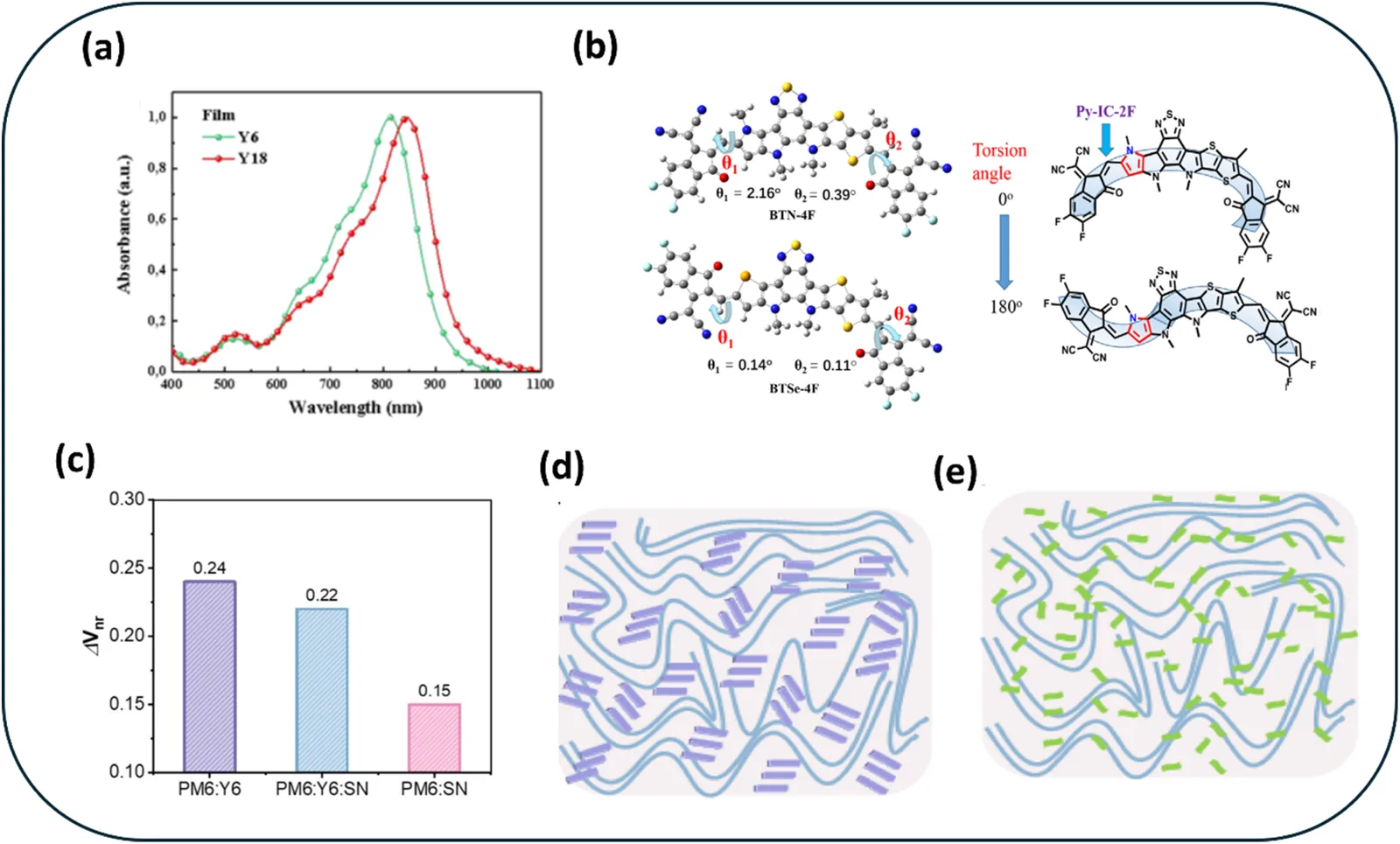
(a) absorption spectra of Y6 and Y18 NFAs, reproduced with permission from ref. 98, copyright 2020, Royal Society of Chemistry; (b) DFT-optimized molecular geometries of BTN-4F and BTSe-4F and corresponding torsional angles, exhibiting ‘S’ and ‘C’ shape geometry, reproduced with permission from ref. 99, copyright 2020, Wiley-VCH GmbH; (c) the non-radiative voltage loss (Δ*V*_nr_) of PM6 and corresponding NFAs, reproduced with permission from ref. 40, copyright 2022, Wiley-VCH GmbH; (d and e) morphology of the binary blends of LC301 and BTA-*m*-S-2Cl, reproduced with permission from ref. 102, copyright 2025, American Chemical Society.

The beneficial influence of nitrogen incorporation was further demonstrated by Luo *et al.*, by developing two asymmetric acceptors, BTSe-4F and BTN-4F, containing selenophene and pyrrole units, respectively. Owing to the stronger electron-donating nature of pyrrole, BTN-4F exhibited elevated energy levels and a pronounced bathochromic shift of ∼40 nm compared to BTSe-4F. More importantly, the two acceptors adopted different molecular conformations, where BTN-4F adopts a favorable “C”-shaped geometry, whereas BTSe-4F exhibits an “S”-shaped conformation with smaller torsional angles, as shown in Fig. 10b. When blended with PM6, the BTN-4F-based devices achieved a higher PCE of 15.82% with a reduced total energy loss of 0.514 eV. In contrast, the PM6:BTSe-4F device delivered a comparatively lower PCE of 13.79% with a higher energy loss of 0.579 eV.^99^

Recently, Li *et al.* developed a series of BTA-based four small-molecule acceptors, BTA-C6, BTA-E3, BTA-E6, and BTA-E9, through side-chain engineering to enhance the photovoltaic performance and mechanical robustness of OSC. Among them, BTA-E3, featuring dynamic hydrogen-bonding ethyl ester side chains, exhibited improved charge transport, molecular packing, and phase separation. As a result, when blended with PM6 polymer donor, the PM6:BTA-E3 based OSCs processed with *o*-xylene achieved an impressive PCE of 19.92% (certified 19.57%), along with enhanced stretchability and thermal stability.^100^ More recently the same group developed a series of narrow-bandgap small-molecule acceptors, namely E3-2Cl, E3-2Br, and E3-2I, through an asymmetric halogen heavy-atom modification strategy to minimize energy losses in OSCs. The heavy-atom effect suppressed molecular vibrations and reorganization energy, reducing non-radiative energy losses and improving the open-circuit voltage. Among them, E3-2Cl-based OSCs achieved a PCE of 20.7% with an exceptionally low energy loss of 0.488 eV. Furthermore, perovskite-organic tandem solar cells incorporating E3-2Cl achieved a PCE of 28.2% (certified 27.5%).^101^

Beyond symmetric core modification, asymmetric molecular design has emerged as another powerful strategy for minimizing voltage losses. Liu *et al.* reported the asymmetric nitrogen-substituted acceptor, SN, which possesses a lower optical bandgap and a red-shifted absorption by ∼40 nm relative to Y6, enabling more NIR light harvesting while maintaining high visible transparency, making it attractive for semi-transparent OSCs. Consequently, the devices based on PM6:SN blends delivered a PCE of 14.3% with an exceptionally ultra-low nonradiative voltage loss of only 0.15 eV, one of the lowest values reported for OSCs and even lower than that of crystalline silicon (c-Si) solar cells (0.18 eV), as shown in Fig. 10c. The remarkable reduction in energy loss was attributed to the enhanced molecular dipole moment induced by the asymmetric molecular architecture. Furthermore, incorporation of SN into ternary PM6:Y6 blends increased the PCE to 17.5% owing to more efficient exciton dissociation, improved charge transport, and enhanced charge extraction.^40^ Recently, Chen *et al.* demonstrated the importance of matching donor and acceptor molecular structures through the development of the NIR-absorbing asymmetric acceptor BTA-*m*-S-2Cl, which contains a benzotriazole central unit and was paired with the BTA-containing polymer donor PBDF-NS. Compared with the BT-based reference acceptor LC301, BTA-*m*-S-2Cl exhibits broader and red-shifted optical absorption, along with a higher LUMO energy level, which is favorable for achieving a high *V*_OC_. More importantly, the two acceptors display markedly different aggregation behaviors during film formation. Whereas LC301 shows a strong tendency toward self-aggregation, leading to coarse phase separation, BTA-*m*-S-2Cl exhibited moderated aggregation that enabled the formation of an optimized nanoscale morphology with appropriate phase-separation length scales (Fig. 10d and e). As a result, the PBDF-NS: BTA-*m*-S-2Cl-based OSCs deliver an outstanding PCE of 18.27%, with a high *V*_OC_ of 0.857 V, a *J*_SC_ of 27.36 mA cm^−2^, and a FF of 77.9%, significantly outperforming the corresponding PBDF-NS:LC301 devices (16.93%).^102^ The molecular architectures and opto-electronic properties were summarized in Fig. 11 and Table 2.

**Fig. 11:**
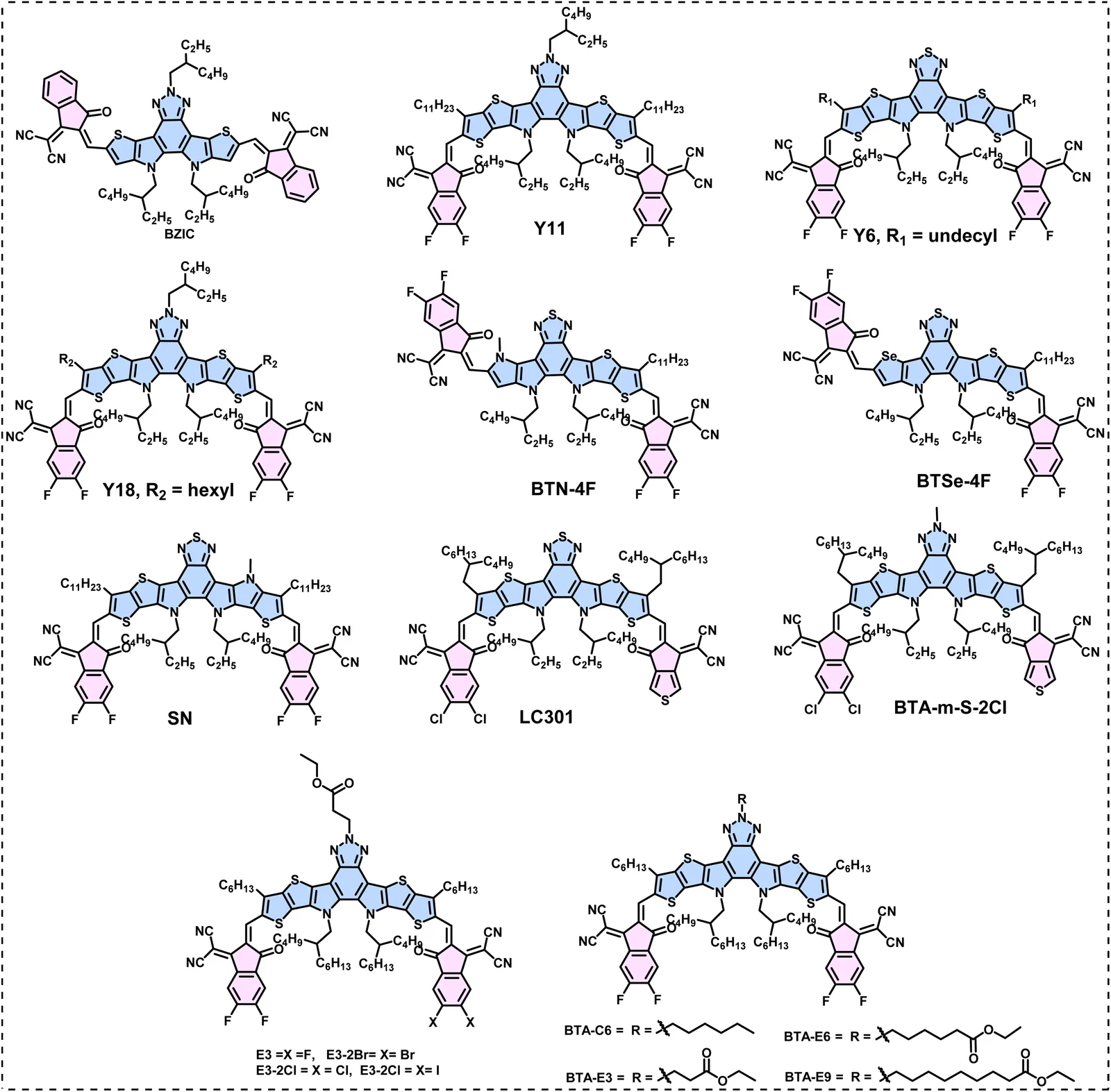
Chemical structures of nitrogen-containing acceptors.

**Table 2:** Photovoltaic properties of nitrogen-containing acceptor molecules discussed in this section

Donor	Acceptor	*J*_SC_ (mA cm^−2^)	*V*_OC_ (V)	FF (%)	PCE (%)	*E* ^opt^_g_ (eV)	HOMO (eV)	LUMO (eV)	*E*_loss_ (eV)	Ref.
HFQx-T	BZIC	12.67	0.84	0.59	6.30	1.45	−5.42	−3.88	0.60	97
PM6	Y11	26.74	0.833	74.33	16.54	1.31	−5.69	−3.87	—	34
PM6	Y18	25.71	0.84	76.5	16.52	1.31	−5.58	−3.91	0.53	98
	Y6	25.20	0.82	76.1	15.71	1.33	−5.66	−4.08	0.58	
PM6	BTN-4F	25.05	0.816	77.3	15.82	1.33	−5.73	−4.15	0.514	99
	BTSe-4F	22.52	0.811	75.4	13.79	1.39	−5.78	−4.16	0.579	
PM6	BTA-C6	27.12	0.83	76.17	17.29	1.30	−5.61	−3.94	—	100
	BTA-E3	28.99	0.85	80.63	19.92	1.32	−5.61	−3.93	0.50	
	BTA-E6	26.74	0.83	73.15	16.27	1.31	−5.60	−3.93	0.51	
	BTA-E9	25.34	0.81	72.63	14.93	1.31	−5.60	−3.94	0.53	
PM6	E3	29.03	0.85	80.56	19.9	1.30	−5.56	−4.11	0.49	101
	E3-2Cl	29.67	0.85	81.44	20.7	1.29	−5.56	−4.09	0.48	
	E3-2Br	29.32	0.86	81.08	20.6	1.29	−5.56	−4.06	0.48	
	E3-2I	28.59	0.87	79.48	19.8	1.29	−5.54	−4.04	0.47	
PM6	SN	25.14	0.82	68.9	14.3	∼1.31	−5.51	−3.82	0.53	40
PBDF-NS	LC301	25.97	0.852	76.5	16.93	1.43	−5.60	−3.85	—	102
	BTA-*m*-S-2Cl	27.36	0.857	77.9	18.27	1.38	−5.56	−3.67	—	

After the emergence of ITIC, A–D–A-type small-molecule acceptors incorporating fused electron-rich central cores have become a widely adopted strategy for high-performance OSCs. A major breakthrough was achieved with the development of Y6, which adopted an A–DA′D–A architecture featuring a benzothiadiazole BT-containing fused core. This molecular design strengthened intramolecular donor–acceptor interactions, promoted efficient charge transfer, and enabled single-junction devices with PCEs exceeding 15%. Subsequent incorporation of pyrrole-containing and BTA-based motifs further improved the optoelectronic properties of Y-series acceptors through enhanced electron-donating capability, favorable energy-level alignment, and reduced steric hindrance associated with *N*-alkyl substituents. These features facilitated stronger π–π stacking and reduced energetic disorder, contributing to the high photovoltaic efficiencies achieved by Y-series derivatives.^103,104^

Collectively, these studies establish nitrogen-containing heterocycles as highly effective structural motifs for simultaneously regulating molecular geometry, electronic structure, aggregation behavior, and energy-loss pathways. In particular, BTA-based core engineering has proven effective in suppressing non-radiative recombination while maintaining favorable donor–acceptor interactions and optimized active-layer morphologies. However, despite these significant advances, Y6-derived acceptors remain subject to intrinsic trade-offs among *V*_OC_, *J*_SC_, and FF, which ultimately limit further improvements in device performance.

To overcome these limitations, increasing attention has been directed toward chalcogen engineering of the fused central core, particularly selenium substitution. Compared with sulphur, selenium possesses a larger atomic radius and higher polarizability, enabling stronger intermolecular interactions, enhanced electronic delocalization, and more pronounced near-infrared absorption.^48^ These characteristics offer an attractive route to simultaneously extend light harvesting, improve charge transport, and optimize molecular packing. Consequently, selenium incorporation has emerged as one of the most successful strategies for advancing Y-series acceptors beyond the performance limits of conventional BT-based systems. For example, Chai *et al.* systematically investigated the influence of chalcogen substitution by developing a series of Y6 derivative acceptors, BPF-4F, BPT-4F, and BPS-4F, incorporating furan (O), thiophene (S), and selenophene (Se) units, respectively, into the fused central core. As illustrated in Fig. 12a, density functional theory calculations revealed that both BPT-4F and BPS-4F adopted more coplanar molecular conformations than the furan-based BPF-4F. This enhanced planarity originates from stabilizing O⋯S and O⋯Se noncovalent interactions, which promotes stronger molecular packing, higher electron mobility, and enhanced FF up to ∼79%. Among the three acceptor materials, the thiophene-based BPT-4F delivered the highest PCE of 16.5%, significantly outperforming the furan-based analogue (12.3%) due to more efficient charge transfer and a more favorable film morphology. Notably, replacement of sulphur with selenium substitution BPS-4F induced a pronounced red-shift in absorption, extending the photon response beyond 950 nm while maintaining comparable external quantum efficiency to the sulfur analogue. However, despite its broadened absorption and increased photocurrent generation, relatively weaker charge-transfer characteristics limited further improvements in *V*_OC_, resulting in a slightly lower PCE (16.1%) than that of BPT-4F.^105^ Further insight into the role of selenium incorporation was provided by Lin *et al.*, through a comparative crystallographic study of the benchmark NFA Y6 and its selenium-substituted analogue CH1007. Single-crystal X-ray diffraction analysis revealed that both materials exhibit pronounced face-to-face π-core interactions governed by S⋯N noncovalent interactions between BT units. These interactions not only stabilize the molecular geometry but also lead to the formation of one-dimensional charge-transport channels that further evolve into interconnected two and three-dimensional packing networks, as shown in Fig. 12b. Notably, replacing sulfur with selenium in CH1007 increased molecular polarizability and extended electronic delocalization without disrupting the favorable packing motifs and extended absorption. As a result, PM6:CH1007:PC_71_BM ternary devices delivered an exceptionally high *J*_SC_ of 27.48 mA cm^−2^ and a PCE of 17.08%.^106^ Yu *et al.* further systematically examined the influence of selenium incorporation in A–DA′D–A-type Y6 derivatives through the synthesis of Y6–Se and Y6–2Se. Owing to the higher polarizability of selenium, both derivatives exhibited bathochromic absorption and enhanced intramolecular charge transfer properties compared to Y6. Structural characterization using grazing-incidence wide-angle X-ray scattering (GIWAXS) revealed reduced π–π stacking distances, indicating strengthened intermolecular interactions and more efficient molecular organization in the solid state. More importantly, resonant soft X-ray scattering (RSoXS) demonstrated that the PM6:Y6–Se blend formed an optimized nanoscale phase-separated morphology with higher domain purity, which facilitated efficient exciton dissociation and charge transport while suppressing charge recombination. Atomic force microscopy (AFM) further confirmed the formation of a smoother active-layer surface, favoring improved interfacial contact and charge collection (Fig. 12c). Consequently, the synergistic combination of enhanced molecular packing, balanced charge transport, and optimized bulk-heterojunction morphology enabled PM6:Y6–Se devices to deliver a PCE of 15.82%, accompanied by improvements in both *J*_SC_ and FF compared with the parent Y6 and Y6–2Se systems.^107^

**Fig. 12:**
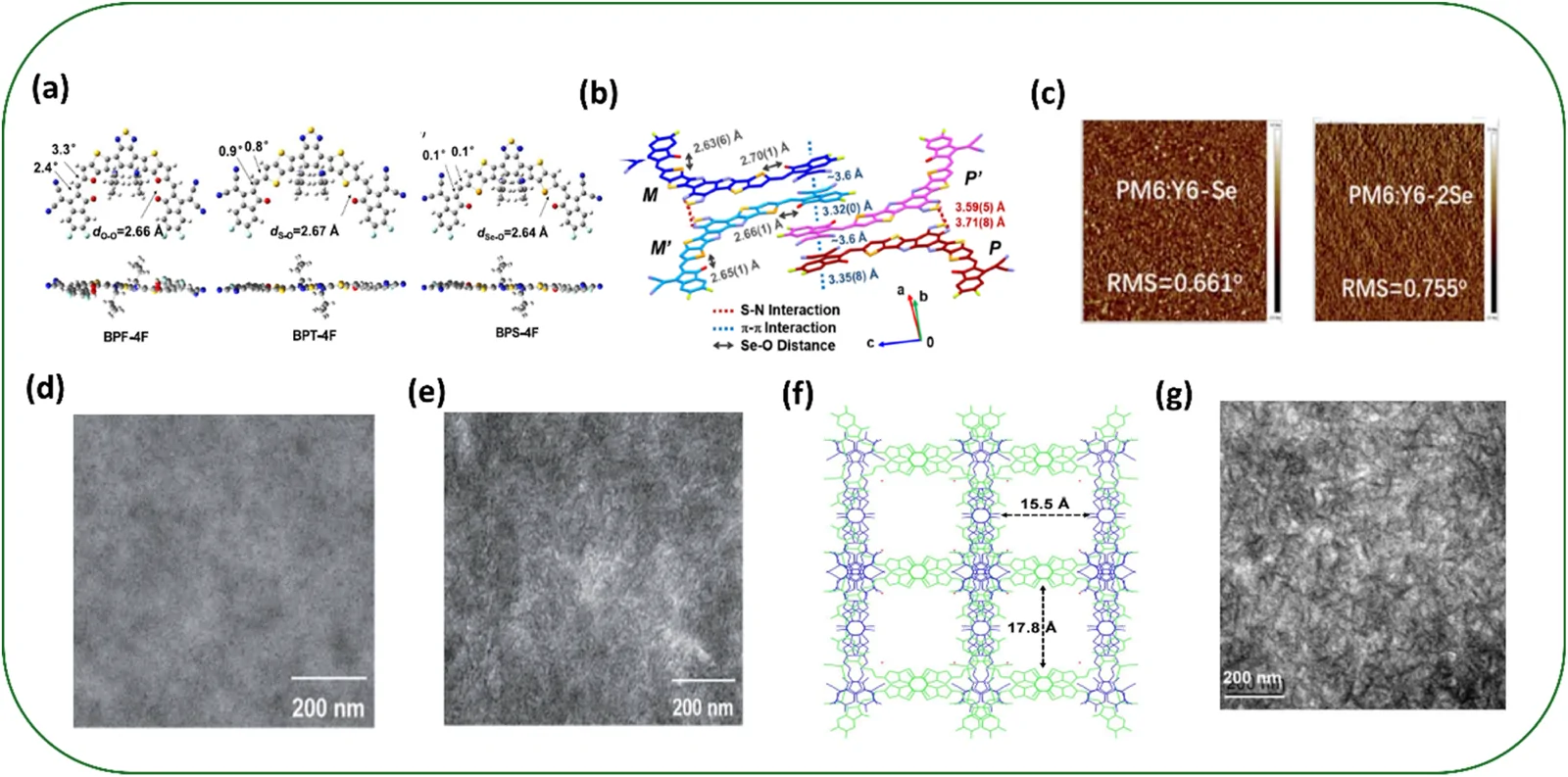
(a) Optimized geometries of BPF-4F, BPT-4F, and BPS-4F molecules, reproduced with permission from ref. 105, copyright 2020, American Chemical Society; (b) single crystal of CH1007, showing S⋯N interactions. reproduced with permission from ref. 106, copyright 2020, American Chemical Society (c) AFM images of PM6: Y6–Se and PM6:Y6-2Se, reproduced with permission from ref. 107, copyright Royal Society of Chemistry; (d and e) TEM images of the optimal PM6:C8TBDT4Cl and PM6:C8T-BDSe4Cl:PM6 blends. Reproduced with permission from ref. 108, copyright Royal Society of Chemistry; (f) single crystal structure of the MQ6 molecule, exhibiting 3D network molecular packing, reproduced with permission from ref. 109, copyright 2021, Wiley-VCH GmbH; (g) TEM image of PM6:A-WSSe-Cl blend exhibiting nano-fibrous network, reproduced with permission from ref. 110, copyright 2021, Wiley-VCH GmbH.

Beyond Y6 and its derivatives, selenium incorporation has been successfully extended to a broader range of NFA architectures. For example, Wan *et al.* developed the selenium-substituted two-dimensional fused-ring electron acceptor, C8T-BDSe4Cl, and systematically compared its properties with its sulfur-based analogue C8T-BDT4Cl. Incorporation of selenium into the central electron-rich core broadened the absorption spectrum, narrowed the bandgap, and strengthened intermolecular interactions. Single-crystal analysis revealed enhanced π–π stacking and tighter three-dimensional packing in C8T-BDSe, which are favorable for efficient charge transport. Importantly, these structural advantages were retained in the corresponding PM6 blend films, leading to a preferred face-on molecular orientation and optimized bulk-heterojunction morphology. Morphological studies using AFM and transmission electron microscopy (TEM) showed that both blends formed smooth surfaces and well-developed interpenetrating nanofibrillar networks, as shown in Fig. 12d and e. Notably, the selenium C8T-BDSe4Cl blend displayed more suitable phase separation and a more continuous nanoscale interpenetrating fibril morphology. Such features facilitated efficient exciton dissociation and charge transport, while the slightly increased aggregation further improved interfacial contact and charge collection. Consequently, the C8T-BDSe4Cl devices achieved a high PCE of 13.5%, with an enhanced *J*_SC_ of 21.79 mA cm^−2^, significantly outperforming the sulfur-based C8T-BDT4Cl devices (12.2%).^108^

The beneficial role of selenium was further highlighted by Tang *et al.*, by developing a series of ladder-type heteroheptacene NFAs, namely, MQ3, MQ5, and MQ6, to elucidate the influence of noncovalent interactions and molecular symmetry on photovoltaic performance. Although MQ3 and MQ5 contain the identical number of selenophene units, the positioning of these units differs substantially, where MQ5 features terminal selenophene rings, whereas MQ3 is flanked by thiophene units. The stronger O⋯Se noncovalent interactions in MQ5, compared with O⋯S interactions present in MQ3, enhanced molecular planarity and electron delocalization, resulting in a bathochromically shifted absorption spectrum, a reduced bandgap, and enhanced charge-carrier transport. As a result, the PCE increased from 13.51% for MQ3 to 15.64% for MQ5. More importantly, both MQ5 and MQ6 adopted favorable acceptor-to-donor (A-to-D) J-aggregate packing motifs and interconnected three-dimensional transport networks that facilitate charge migration. Notably, the asymmetric heteroheptacene core of MQ6 generated a larger molecular dipole moment, resulting in a shorter π–π stacking distance (3.48 Å), as shown in Fig. 12f, than that of MQ5 (3.55 Å). The tighter molecular packing strengthened intermolecular electronic coupling and enhanced carrier mobility while preserving the advantageous three-dimensional packing framework. Consequently, PM6:MQ6 devices delivered a superior PCE of 16.39%, accompanied by a high *J*_SC_ of 24.62 mA cm^−2^, an FF of 75.66%, and a *V*_OC_ of 0.88 V.^109^

Beyond symmetric core modification, asymmetric selenium incorporation has emerged as a powerful molecular design strategy for simultaneously optimizing molecular packing, charge transport, and morphology. In this context, Yang *et al.* systematically investigated the synergetic effects of molecular symmetry and selenophene substitution by developing a series of A–D_1_A′D_2_–A-type SMAs, namely S-YSS-Cl, A-WSSe-Cl, and S-WSeSe-Cl, by varying the Se units on the core moiety. Increasing selenium incorporation progressively red-shifted absorption, enhanced molecular crystallinity, and improved electron mobility owing to strengthened intermolecular interactions and tighter π–π stacking. Among the three NFAs, the disymmteric A-WSSe-Cl exhibited the most balanced optoelectronic properties. The introduction of both molecular asymmetry and strategically positioned selenophene units not only reinforced intermolecular π–π interactions but also additional S⋯N noncovalent interactions involving the central benzothiadiazole unit, leading to more ordered three-dimensional interpenetrating charge-transport networks. Morphological characterization by AFM and TEM further revealed that both selenium-incorporated blends possessed an optimized nanofiber-like interpenetrating network and appropriate multi-length-scale phase separation (Fig. 12g), indicating excellent morphological compatibility between the donor and acceptor, particularly PM6:A-WSSe-Cl. Consequently, PM6:A-WSSe-Cl devices delivered a high PCE of 17.51% with an enhanced FF, outperforming both symmetric counterparts.^110^

Qi *et al.* further demonstrated the potential of selenium-containing fused π-cores by designing BzS-based NFAs, mBzS-4F and EHBzS-4F, in which a weakly electron-withdrawing benzotriazole unit was combined with a strongly electron-donating selenophene to form an extended π-core. This molecular design significantly enhanced intramolecular charge transfer and intra/intermolecular interactions, enabling both acceptors to exhibit absorption edges ∼1000 nm (Fig. 13a). Notably, side-chain engineering on the benzotriazole unit played a critical role in regulating blend morphology. The linear *N*-alkyl substituent in mBzS-4F facilitated efficient molecular organization and balanced charge transport, enabling the blend devices to achieve a high PCE of 17.02%, together with an exceptionally high *J*_SC_ of 27.72 mA cm^−2^ and a remarkably low energy loss of 0.446 eV. In contrast, EHBzS-4F, containing branched *N*-alkyl side chains, exhibited inferior photovoltaic performance with a PCE of 15.94%.^111^

**Fig. 13:**
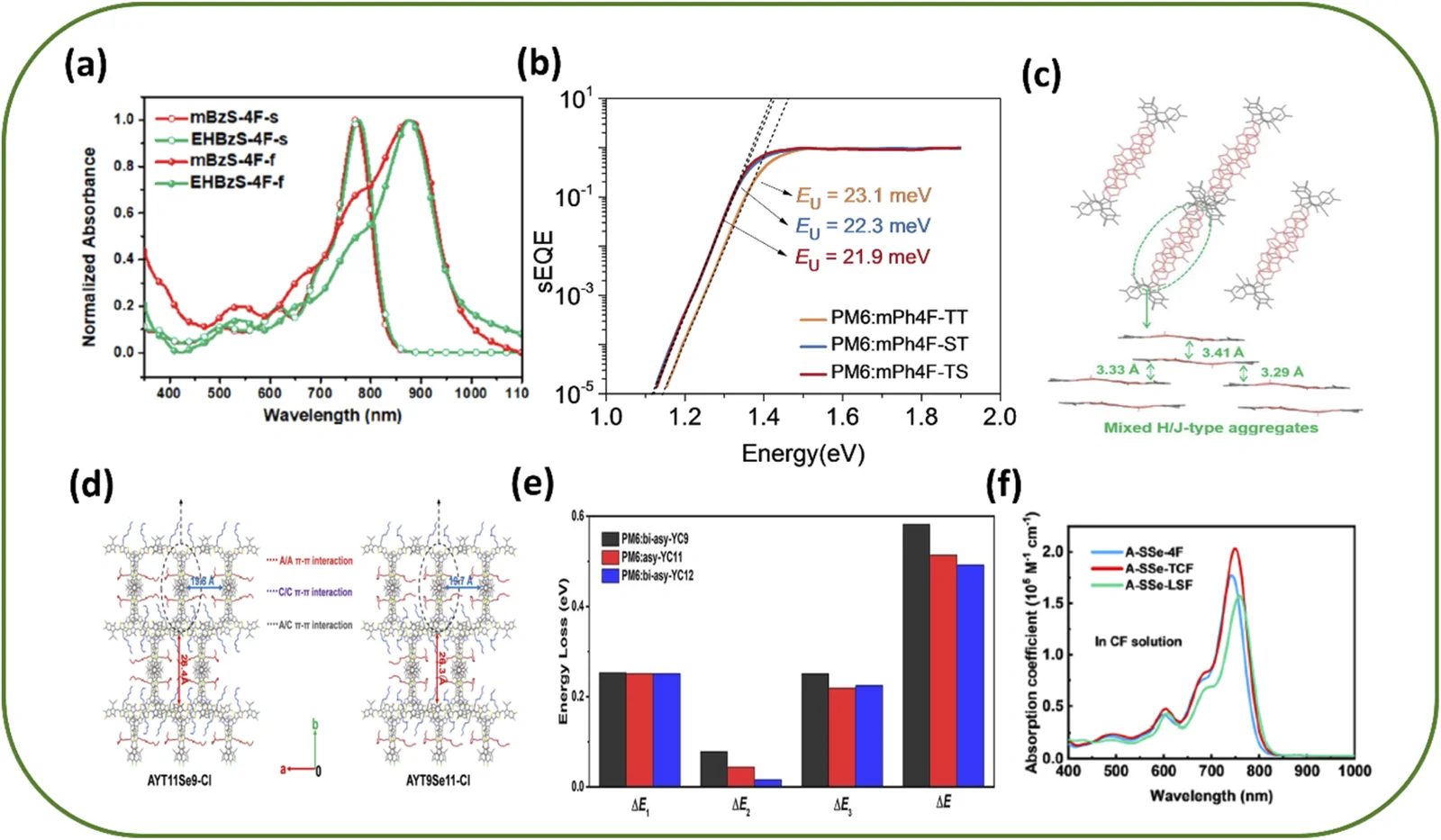
(a) UV-vis absorption spectra of mBzS-4F and EHBzS-4F, showing absorption edges extending to ∼1000 nm, reproduced with permission from ref. 111. Copyright 2021, American Chemical Society. (b) Urbach energy plots of PM6:mPh4F-TT, PM6: mPh4F-ST, and PM6: mPh4F-TS blend films, reproduced with permission from ref. 112. Copyright 2022, Wiley-VCH GmbH. (c) molecular packing of MQX4 illustrating mixed H/J-type aggregation, reproduced with permission from ref. 114. Copyright 2023 SIOC, CAS Shanghai, & Wiley-VCH GmbH. (d) Comparison of the molecular packing along the *c*-axis in the single-crystal structures of AYT11Se9-Cl and AYT9Se11-Cl, reproduced with permission from ref. 115. Copyright 2023, Wiley-VCH GmbH. (e) Radiative and non-radiative energy losses of the bi-asy-YC12-based photovoltaic devices, reproduced with permission from ref. 116. Copyright 2023, Wiley-VCH GmbH. (f) Absorption coefficients of A-SSe-4F, A-SSe-TCF, and A-SSe-LSF in chloroform solution. Reproduced with permission from ref. 117. Copyright 2025, American Chemical Society.

Beyond the extent of selenium, the positional placement of selenium atoms within the central π-core has emerged as a crucial factor governing the optoelectronic properties, molecular packing, and photovoltaic performance of NFA. To elucidate these effects, Zhang *et al.* designed two positional isomers, mPh4F-TS and mPh4F-ST, in which selenophene units were incorporated at the outer and inner positions of the fused π-core, respectively, and compared their properties with those of the thiophene-based parent acceptor mPh4F-TT. Among the three materials, mPh4F-TS, featuring selenium atoms at the outer positions of the fused backbone, exhibited the most pronounced bathochromic absorption, the highest molecular rigidity, and the most compact intermolecular packing. These favorable structural characteristics translated into superior device performance when blended with PM6. As shown in Fig. 13b, PM6:mPh4F-TS devices exhibited reduced energy loss and Urbach energy, broadened and enhanced external quantum efficiency (EQE) response, suppressed charge recombination, and more efficient charge transport and extraction. Collectively, these effects mitigated the conventional trade-offs among *V*_OC_, *J*_SC_, and FF, enabling simultaneous improvements in all three photovoltaic parameters. As a result, PM6:mPh4F-TS devices achieved an outstanding PCE of 18.05%, surpassing those based on mPh4F-ST (17.57%) and mPh4F-TT (16.88%).^112^ A similar positional effect was observed by Gao *et al.*, who synthesized the acceptors S9TBO-4F and BS3TSe-4F through regioselective replacement of sulfur with selenium at different locations within the π-conjugated core of BP4T-4F. The asymmetric selenium incorporation in BS3TSe-4F induced a pronounced red shift in absorption and increased the dielectric constant, thereby facilitating exciton dissociation and charge transport while simultaneously reducing radiative recombination losses. Owing to these synergistic advantages, D18:BS3TSe-4F planar heterojunction devices delivered an enhanced PCE of 18.48%, outperforming the corresponding S9TBO-4F-based devices (17.71%).^113^ The importance of selenium-mediated noncovalent interactions was further demonstrated by Tao *et al.*, who developed a new class of nitrogen-bridged, sp^3^-carbon-free ladder-type heteroarene acceptors, MQX-2 and MQX-4, end-capped with thiophene (BTPS) and selenophene (BTPSe), respectively. Compared with MQX-2, the stronger O⋯Se noncovalent interactions present in MQX-4 significantly altered the aggregation behavior and molecular packing mode. Whereas MQX-2 predominantly formed H-type aggregates, MQX-4 exhibited a mixed H/J-aggregate character, resulting in a more multidimensional molecular stacking architecture, as shown in Fig. 13c. This transition generated additional charge-hopping pathways and enhanced intermolecular electronic coupling, thereby facilitating more efficient electron transport. Consequently, the electron mobility of MQX-4 blend films (1.80 × 10^−5^ cm^2^ V^−1^ s^−1^) was more than twice that of MQX-2 (7.70 × 10^−6^ cm^2^ V^−1^ s^−1^). The improved charge-transport characteristics translated directly into enhanced photovoltaic performance, with PBDB-T blend devices achieving a PCE of 9.72%, significantly higher than that of the corresponding MQX-2-based devices (7.09%).^114^

While precise positioning of selenium atoms within the fused backbone provides an effective means of regulating molecular packing and electronic structure, further optimization can be achieved through side-chain engineering. By modulating steric effects, intermolecular interactions, and phase-separation behavior, side chains offer an additional degree of freedom for fine-tuning the morphology and charge-transport properties of selenium-containing acceptors. In this context, Zhao *et al.* developed two double-asymmetric regioisomeric small-molecule acceptors, AYT11Se9-Cl and AYT9Se11-Cl, by introducing asymmetric *n*-nonyl/*n*-undecyl side chains onto an asymmetric selenophene-centered A–D_1_A′D_2_–A fused backbone. Although the two regioisomers exhibited nearly identical optical band gaps and frontier energy levels, subtle differences in side-chain positioning resulted in markedly different solid-state organizations and photovoltaic characteristics. Crystallographic analysis revealed that AYT9Se11-Cl, in which the *n*-undecyl chain occupies the β-position of the selenophene unit, adopts a more compact and ordered intermolecular packing arrangement than AYT11Se9-Cl, as shown in Fig. 13d. The enhanced molecular ordering promoted stronger crystallinity and higher electron mobility in the neat film. These advantages were further reflected in the corresponding PM6 blend films, where AYT9Se11-Cl exhibited a more favorable morphology characterized by a pronounced face-on orientation and well-defined fibrous phase separation. Consequently, when blended with PM6 polymer donor, the AYT9Se11-Cl devices achieved a high PCE of 18.12%, surpassing that of AYT11Se9-Cl (17.52%) and representing one of the highest efficiencies reported for selenium-containing small-molecule acceptors in binary BHJ-OSCs.^115^ The importance of side-chain regulation was further highlighted by Fan *et al.*, who demonstrated that dual-asymmetric side-chain engineering provides a powerful strategy for simultaneously optimizing molecular packing, morphology, and energy-loss characteristics in Y-series acceptors, and developed a series of acceptors comprising the mono-asymmetric asy-YC11 and the dual-asymmetric bi-asy-YC9 and bi-asy-YC12. All three acceptors share the same asymmetric D_1_–A–D_2_ fused core constructed from thieno[3,2-*b*]thiophene (TT) and selenopheno[3,2-*b*]thiophene (SeT) units but differ in the arrangement of unidirectional side chains on the TT segment. This design enabled precise control over intermolecular interactions, crystallinity, molecular packing, and near-infrared absorption. Among the three acceptors, bi-asy-YC12 exhibited the most favorable solid-state organization. As a result, PM6:bi-asy-YC12 blend films formed a highly ordered morphology with appropriate phase separation and enhanced π–π stacking, facilitating efficient charge transport while suppressing non-radiative energy losses (Fig. 13e) by achieving a PCE of 17.16%, accompanied by simultaneous enhancements in both *V*_OC_ and *J*_SC_. Benefiting from its strong NIR absorption and well-matched energy levels, bi-asy-YC12 was subsequently incorporated as a guest acceptor into PM6:L8BO blends, enabling annealing-free ternary devices to achieve a champion PCE of 19.23%, among the highest efficiencies reported for ternary organic solar cells.^116^ Recently, Yin *et al.* extended the concept of asymmetry beyond side-chain regulation by simultaneously engineering both the fused backbone and terminal acceptor groups. This dual-asymmetric molecular design strategy led to the development of A-SSe-TCF and A-SSe-LSF, whose properties were systematically compared with those of the mono-asymmetric analogue A-SSe-4F. Introducing asymmetry into both the core and terminal units broadened the visible-to-near-infrared absorption profile, increased half-molecule dipole moment differences, and reduced non-radiative energy losses, thereby creating favorable conditions for simultaneously enhancing *V*_OC_ and *J*_SC_. Among the three acceptors, A-SSe-TCF exhibited the most balanced optoelectronic and morphological characteristics. Owing to enhanced intermolecular aggregation induced by terminal-group chlorination, A-SSe-TCF displayed a more red-shifted absorption profile, a higher absorption coefficient (Fig. 13f), and stronger molecular stackability than the mono-asymmetric reference A-SSe-4F. More importantly, the corresponding PM6:A-SSe-TCF blend formed a highly optimized active-layer morphology featuring suitable phase separation, improved vertical phase distribution, and enhanced π–π stacking order, more exciton dissociation, and lower trap-assisted recombinations. Consequently, the A-SSe-TCF devices achieved an impressive PCE of 18.53%, outperforming PM6:A-SSe-4F devices (17.75%). Furthermore, the excellent compatibility of A-SSe-TCF with high-performance acceptor systems enabled its application as a third component in ternary devices, resulting in a champion PCE of 19.73%.^117^ The chemical structures and photovoltaic properties of the above-discussed acceptors are summarized in Table 3 and Fig. 14.

**Table 3:** Photovoltaic properties of the above-discussed acceptor molecules

Donor	Acceptor	*J*_SC_ (mA cm^−2^)	*V*_OC_ (V)	FF (%)	PCE (%)	*E* ^opt^_g_ (eV)	HOMO (eV)	LUMO (eV)	*E*_loss_ (eV)	Ref.
SZ5	BPF-4F	22.1	0.845	66.2	12.3	1.36	−5.58	−3.98	0.56	105
	BPT-4F	24.7	0.853	78.0	16.5	1.36	−5.59	−4.00	0.56	
	BPS-4F	25.3	0.822	77.3	16.1	1.29	−5.54	−4.00	0.56	
PM6	CH1007 + PC_71_BM	27.48	0.822	75.61	17.08	1.30	−5.59	−3.97	0.48	106
	Y6 + PC_71_BM	25.80	0.841	75.70	16.41	1.39	−5.60	−3.94	0.55	
PM6	Y6-Se	25.47	0.82	75.00	15.82	1.31	−5.55	−3.83	—	107
	Y6-2Se	24.32	0.83	70.00	14.62	1.34	−5.58	−3.84	—	
	Y6	24.80	0.83	74.00	15.35	1.36	−5.61	−3.84	—	
PM6	C8T-BDT4Cl	19.65	0.86	72.32	12.21	1.40	−5.75	−3.92	—	108
	C8T-BDSe4Cl	21.79	0.85	72.71	13.50	1.39	−5.71	−3.96	—	
PM6	MQ3	22.19	0.91	66.92	13.51	1.37	−5.63	−3.94	—	109
	MQ5	24.48	0.86	74.31	15.64	1.34	−5.59	−3.95	—	
	MQ6	24.62	0.88	75.66	16.399	1.35	−5.61	−3.95	—	
PM6	S-YSS-Cl	25.85	0.86	75.28	16.73	1.34	−5.71	−3.85	—	110
	A-WSSeCl	26.58	0.85	77.50	17.51	1.31	−5.70	−3.86	—	
	S-WseSe-Cl	26.35	0.82	73.41	16.01	1.30	−5.66	−3.88	—	
PM6	mBzS-4F	27.72	0.80	76.35	17.02	1.25	−5.60	−3.90	0.446	111
	EHBzS-4F	27.58	0.82	70.07	15.94	1.24	−5.61	−3.86	—	
PM6	mPh4F-TT	25.00	0.86	76.20	16.88	1.36	−5.63	−3.87	—	112
	mPh4F-ST	26.83	0.82	77.90	17.57	1.31	−5.64	−3.92		
	mPh4F-TS	27.39	0.82	78.50	18.05	1.30	−5.61	−3.92		
D18	S9TBO-F	28.19	0.83	74.93	17.71	1.29	−5.61	−3.84	0.547	113
	BS3TSe-4F	29.40	0.82	75.94	18.48	1.28	−5.60	−3.86	0.540	
PBDB-T	MQX-2	14.02	0.87	58.39	7.09	1.37	−5.56	−3.90	—	114
	MQX-4	17.91	0.83	65.52	9.72	1.34	−5.51	−3.91		
PM6	AYT11Se9-Cl	26.86	0.84	77.47	17.52	1.33	−5.69	−3.89	—	115
	AYT9Se11-Cl	27.40	0.84	78.43	18.12	1.33	−5.68	−3.88		
PM6	bi-asy-YC9	22.14	0.811	58.20	10.45	∼1.30	−5.56	−4.01	0.582	116
	asy-YC11	26.74	0.849	72.70	16.52	∼1.30	−5.53	−4.01	0.514	
	bi-asy-YC12	26.39	0.871	74.30	17.16	∼1.30	−5.53	−4.01	0.492	
PM6	A-SSe-4F	27.22	0.84	77.36	17.75	1.32	−5.70	−3.92	0.574	117
	A-SSe-TCF	27.86	0.85	78.14	18.53	1.31	−5.72	−3.96	0.549	
	A-SSe-LSF	26.43	0.85	73.95	16.68	1.31	−5.66	−3.91	0.535	

**Fig. 14:**
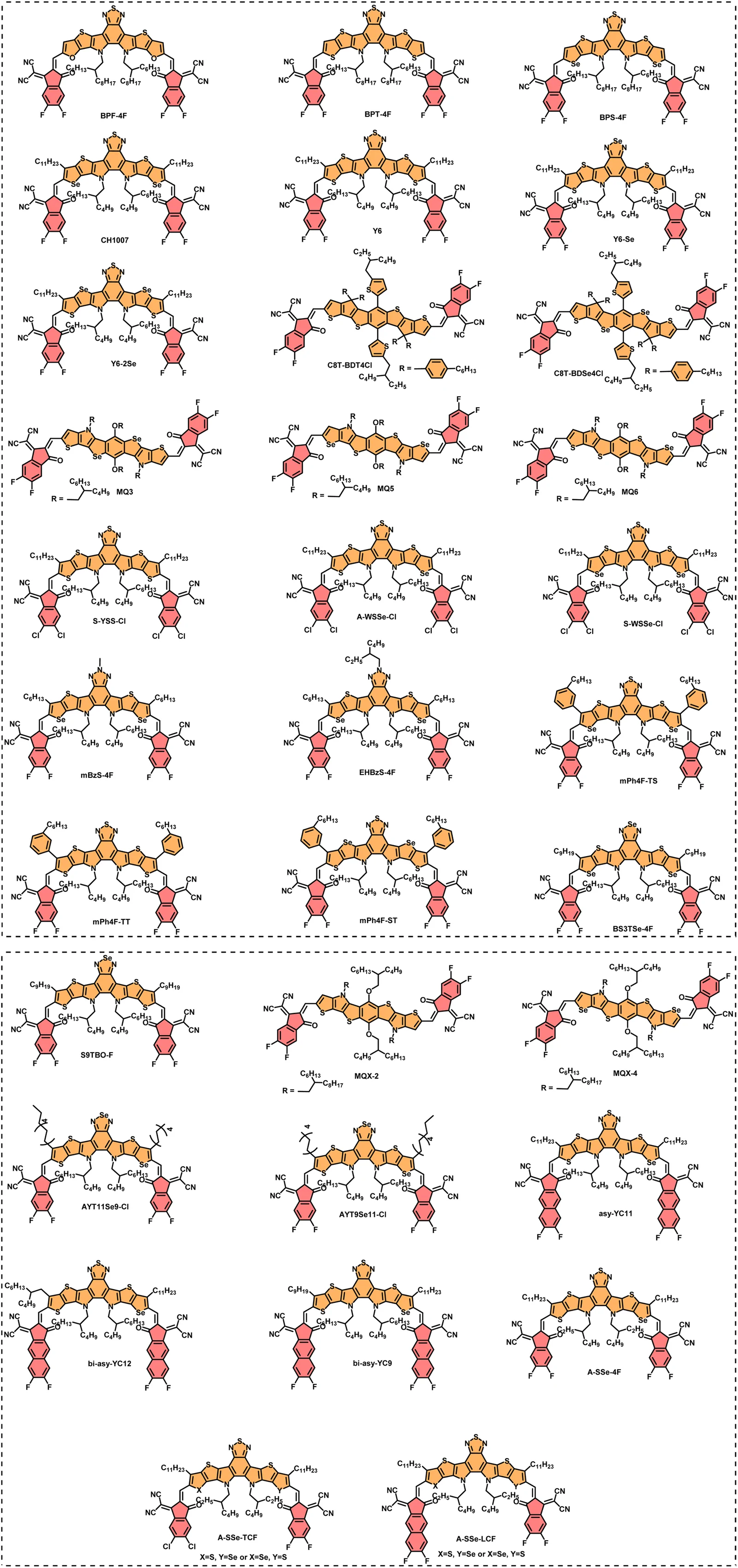
Chemical structures of the above-discussed acceptor molecules.

### Effect of halogen side chain on BDT-based NFAs

In addition to fused-core engineering, side-chain modification has emerged as an effective approach for further improving the performance of Y-series acceptors. In particular, the introduction of aromatic substituents onto the peripheral thiophene units of the BDT-based fused backbone can extend π-conjugation along the lateral direction, providing an additional means of tuning the optoelectronic properties of NFAs. Such modifications influence ICT, molecular energy levels, and optical absorption properties. Beyond electronic modulation, these quasi-conjugated side chains significantly influence molecular conformation and intermolecular interactions by altering steric hindrance, backbone planarity, and dihedral angles, thereby governing aggregation behavior and solid-state packing.^118–120^ Among the various side-chain engineering strategies, halogen functionalization has attracted considerable attention because it simultaneously modifies both electronic and morphological properties. In addition, due to their strong electron-withdrawing nature, halogen atoms can regulate frontier molecular orbital energies, molecular dipole moments, and intermolecular interactions. As a result, halogenated side chains have proven highly effective for balancing charge generation, charge transport, and energy losses, while often improving the morphological stability of the active layer. These advantages have made halogen side-chain engineering an important design strategy for developing high-performance BT-based NFAs. For example, Tan *et al.* systematically investigated the effect of positional chlorine substitution on thiophene side chains by developing a series of NFAs, namely BTIC-4Cl-T, BTIC-4Cl-TCl-γ, and BTIC-4Cl-TCl-β. Their study demonstrated that the position of the chlorine substitution plays a crucial role in regulating molecular geometry, energy levels, and solid-state packing. Chlorination progressively increased steric hindrance, while substitution at the β-position induced greater backbone twisting, leading to a gradual blue shift in the absorption spectra and an upshifted LUMO energy level. These changes effectively reduced energy loss and enhanced the *V*_OC_. Single-crystal structural analysis further revealed that BTIC-4Cl-TCl-β adopts a distinctive quasi-three-dimensional packing network, arising from precise positional chlorination and reinforced intermolecular interactions. Importantly, GIWAXS measurements confirmed that key packing motifs observed in the blend films, while the dominant face-on molecular orientation facilitated efficient vertical charge transport. As a result, BTIC-4Cl-TCl-β-based devices exhibited a high *V*_OC_ of 0.86 V and achieved the highest PCE of 15.65%, where BTIC-4Cl-T and BTIC-4Cl-TCl-γ achieved a PCE of 10.86.% and 14.35% PCE only.^121^ Apart from chlorination, fluorinated side chains have been widely explored as an effective strategy for regulating molecular packing and charge extraction. For instance, Chong *et al.* developed two Y-series acceptors, BTP-Th and BTP-FTh, featuring two-dimensional side (2D) chains with different degrees of fluorination. Although the incorporation of bulky 2D side chains increased steric hindrance and partially disrupted molecular packing and phase separation, fluorination effectively compensated for these unfavorable effects by enhancing molecular crystallinity and electron mobility without significantly altering the optical properties. Consequently, PTQ10:BTP-FTh blended devices achieved a PCE of 17.16%, compared with 15.89% for the corresponding non-fluorinated PTQ10:BTP-Th blend devices. To further optimize the active-layer morphology, IDIC was introduced as a secondary acceptor and DTT as a volatilizable solid additive. These highly crystalline components promoted molecular ordering and refined the nanoscale phase-separated morphology, leading to more efficient charge extraction and suppressed charge recombination. As a result, the FF increased progressively from 76.7% for the binary device to 78.6% after incorporating IDIC. Simultaneously, the optimized ternary blend exhibited an improved *V*_OC_ of 0.870 V and a high *J*_SC_ of 27.17 mA cm^−2^, ultimately delivering a record PCE of 19.05%.^122^ Li *et al.* developed a low-cost polymer donor, PTQ10, through a simple two-step synthesis with a high overall yield of 87.4%, which exhibited broad absorption in the 450–620 nm range, a medium optical bandgap of 1.92 eV, and a deep HOMO energy level of −5.54 eV. As a result, the PTQ10:IDIC blend achieved a PCE of 12.70%, while inverted devices reached 12.13%. Notably, the devices demonstrated thickness-insensitive photovoltaic performance over a wide active-layer thickness range of 100–300 nm, highlighting the potential of PTQ10 for low-cost, large-area polymer solar cell fabrication.^123^

Beyond fluorination and chlorination, heavier halogens have also been employed to exploit steric effects more effectively while improving device stability. For instance, Fan *et al.* developed two brominated acceptors, BTP-Br and BTP-TBr, featuring distinct side-group architectures. Compared with the outwardly brominated BTP-Br, the incorporation of a 2-bromothienyl side group in BTP-TBr introduced greater steric hindrance, resulting in increased side-chain torsion and a more twisted molecular backbone. Interestingly, despite the reduced molecular planarity, BTP-TBr retained a narrow optical bandgap and low energy loss, demonstrating that carefully controlled backbone distortion can be beneficial for balancing photovoltaic parameters. Consequently, xylene-processed devices based on BTP-TBr achieved an impressive PCE of 18.3%, among the one of the highest reported for OSCs processed from non-halogenated solvents. More importantly, the enhanced steric hindrance effectively suppressed excessive molecular aggregation, resulting in significantly improved morphological stability during device operation.^124^

Beyond the halogen substituent, more refined control over molecular packing and device stability can be achieved through precise regulation of side-chain geometry. In particular, subtle variations in the position of halogen atoms or the alkyl substituents can significantly alter molecular conformation, intermolecular interactions, and aggregation behavior, thereby influencing photovoltaic performance. For example, Fan *et al.* demonstrated that local isomerization of chlorinated thienyl side groups provides an effective means of fine-tuning molecular geometry and morphology. By systematically varying the chlorine substitution position on the thienyl substituents, they developed three isomeric acceptors, namely 2-ClTh, 3-ClTh, and 4-ClTh. This subtle structural modification enabled precise control over both side-group and terminal-group torsion angles, as well as the associated noncovalent interactions with the fused-ring backbone. As a result, significant differences in molecular conformation, aggregation behavior, and morphological stability were observed among the three isomers. Notably, 3-ClTh exhibited an optimal balance between side-group and terminal-group torsions, leading to moderated molecular packing that effectively suppressed excessive aggregation while maintaining efficient charge-transport pathways. Consequently, D18-Cl:3-ClTh devices achieved an impressive PCE of 18.5% together with a remarkably low energy loss of 0.528 eV. More importantly, the optimized molecular conformation imparted exceptional photothermal stability. Under continuous maximum-power-point tracking at 65 °C under one-sun illumination, the 3-ClTh-based devices retained 72.2% of their initial efficiency after approximately 400 h of aging, significantly outperforming the corresponding 2-ClTh (62.0%) and 4-ClTh (47.7%) devices, as shown in Fig. 16a. The enhanced operational stability was attributed to improved conformational and morphological robustness, which effectively suppressed excessive aggregation and morphology evolution under thermal and photoinduced stress.^125^ The effects of halogen substitution can also be further modulated through the geometry of the alkyl chains attached to the side-chain thiophene units. Tan *et al.* investigated this concept by designing two chlorinated NFAs, BTIC-TCl-b and BTIC-TCl-l, which differ only in the configuration of the alkyl substituents on the side-chain thiophene rings. Interestingly, replacing the linear alkyl chains in BTIC-TCl-l with a branched architecture in BTIC-TCl-b resulted in enhanced molecular planarity, reduced the π–π stacking distance, and decreased the effective molecular frame size. Single-crystal analysis further revealed that this structural evolution transformed the molecular packing from a quasi-three-dimensional arrangement into a more compact and interconnected three-dimensional network, as illustrated in Fig. 16b. The resulting tighter packing facilitated more efficient charge-transport pathways and promoted stronger intermolecular electronic coupling. Consequently, BTIC-TCl-b exhibited enhanced charge-carrier mobilities, more efficient exciton dissociation, and reduced bimolecular recombination compared to its linear-chain counterpart. These advantages translated into enhanced charge generation and collection, leading to simultaneous improvements in *J*_SC_ and FF. As a result, PBDB-TF : BTIC-TCl-b devices achieved an outstanding PCE of 16.17%, surpassing the 15.49% obtained for the corresponding BTIC-TCl-l-based devices.^126^

Beyond the geometry of alkyl substituents, the positional placement of fluorine atoms on two-dimensional side chains provides another powerful means of regulating molecular organization and device stability. Yang *et al.* demonstrated this concept by developing two isomeric Y-series acceptors, 4FY and 2FY, which differ only in the fluorine substitution position on the hexylphenyl side chains. Although the two acceptors possess nearly identical conjugated backbones, frontier molecular orbital energy levels, and backbone geometries, subtle differences in side-chain conformation give rise to markedly different intermolecular interactions and solid-state properties. Owing to the formation of intramolecular C–H⋯F noncovalent interactions, 4FY adopts a diagonally extended and conformationally locked side-chain arrangement, whereas 2FY exhibits a more horizontally oriented configuration. This structural distinction increases the molecular dipole moment of 4FY and strengthens intermolecular interactions, resulting in enhanced crystallinity and denser molecular packing. The improved molecular ordering not only benefited charge transport but also significantly enhanced thermal stability. As shown in Fig. 16c, exhibited a substantially higher glass-transition temperature (*T*_g_) of 143.6 °C compared with 100.8 °C for 2FY, indicating greater resistance to morphology evolution under thermal stress. Consequently, PTQ10:4FY devices achieved a PCE of 16.4%, benefiting from more efficient exciton dissociation, balanced charge transport, and improved donor–acceptor compatibility. More importantly, the enhanced crystallinity and elevated *T*_g_ imparted superior morphological robustness, allowing PTQ10:4FY devices to retain 88.4% of their initial efficiency after thermal aging at 85 °C for 360 h, whereas PTQ10:2FY devices retained only 79.0%.^127^ In addition to positional effects, the extent of fluorination on side chains has also been shown to profoundly influence intermolecular electrostatic interactions and active-layer morphology. Chen *et al.* investigated this effect by synthesizing two acceptors, BTP-F0 and BTP-F5, with different degrees of side-chain fluorination. As illustrated in Fig. 16d, both theoretical calculations show that increasing fluorine content raises the average electrostatic potential (ESP) and strengthens ESP-driven intermolecular interactions. As a consequence, BTP-F5 exhibited stronger molecular packing, enhanced crystallinity, higher domain purity, and reduced domain spacing relative to BTP-F0, resulting in a more favorable multiscale bulk-heterojunction morphology. Further insight was obtained from film-depth-dependent light absorption spectroscopy (FLAS), which revealed that BTP-F5-containing blends possess a more desirable vertical phase distribution characterized by a thin polymer-rich layer near the bottom electrode and an acceptor-rich region near the film surface. Such a vertical composition gradient facilitates exciton diffusion and charge separation while simultaneously promoting charge transport and collection through an enhanced built-in potential. Together with the optimized nanoscale morphology, these features suppressed bimolecular recombination and enabled more balanced carrier transport. Consequently, BTP-F5-based binary devices achieved a PCE of 17.3%, outperforming the corresponding BTP-F0 devices (16.1%). Moreover, incorporation of BTP-F5 into the ternary system further increased the PCE to 19.2%.^128^

Overall, these studies collectively demonstrate that halogen functionalization on side chains is not merely an electronic modification but a powerful structural design tool that governs molecular conformation, electrostatic interactions, and morphological stability. Precise control over halogen type, substitution position, and side-chain geometry enables fine-tuning of aggregation behavior and charge-transport pathways, allowing simultaneous optimization of efficiency and operational stability. These insights establish halogenated side-chain engineering as a central strategy for the rational design of next-generation Y6-type non-fullerene acceptors for high-performance and durable organic solar cells. The key photovoltaic parameters of the molecules discussed in this section are summarized in Table 4 and the chemical structures are shown in Fig. 15.

**Table 4:** Photovoltaic properties of side chain halogenated acceptors

Donor	Acceptor	*J*_SC_ (mA cm^−2^)	*V*_OC_ (V)	FF (%)	PCE (%)	*E* ^opt^_g_ (eV)	HOMO (eV)	LUMO (eV)	*E*_loss_ (eV)	Ref.
PBDB-TF	BTIC-4Cl-T	18.28	0.81	72.62	10.86	1.35	−5.42	−3.89	0.620	121
	BTIC-4Cl-TCl-γ	23.91	0.80	75.24	14.35	1.35	−5.46	−3.91	0.628	
	BTIC-4Cl-TCl-β	24.30	0.86	74.61	15.65	1.36	−5.43	−3.89	0.602	
PTQ10	BTP-Th	24.38	0.901	72.4	15.89	1.33	−5.75	−3.96	—	122
	BTP-FTh	26.33	0.849	76.7	17.16	1.32	−5.80	−4.04	—	
PTQ10	IDIC	17.81	0.969	73.60	12.70	—	−5.74	−3.90	—	123
PTzBl-dF	BTP-Br	25.7	0.752	69.1	13.3	1.429	−5.78	−4.00	0.668	124
	BTP-TBr	27.5	0.845	78.8	18.3	1.401	−5.76	−3.88	0.553	
D18	2ClTh	26.7	0.857	75.6	17.3	—	−5.73	−4.32	0.55	125
	3ClTh	26.9	0.891	77.0	18.5	—	−5.71	−4.29	0.52	
	4ClTh	24.9	0.924	72.7	16.7	—	−5.73	−4.28	0.51	
PBDB-TF	BTIC-TCl-l	23.84	0.88	73.62	15.49	1.37	−5.75	−3.92	—	126
	BTIC-TCl-b	24.29	0.88	75.78	16.17	1.36	−5.70	−3.90	—	
PTQ10	2FY	22.3	0.915	71.0	14.5	1.38	−5.63	−3.94	0.520	127
	4FY	23.8	0.885	77.8	16.4	1.35	−5.63	−3.93	0.521	
PM1	BTP-F0	26.2	0.863	71.0	16.1	∼1.37	−5.44	−4.07	—	128
	BTP-F5	26.9	0.855	75.1	17.3	∼1.37	−5.50	−4.08	—	

**Fig. 15:**
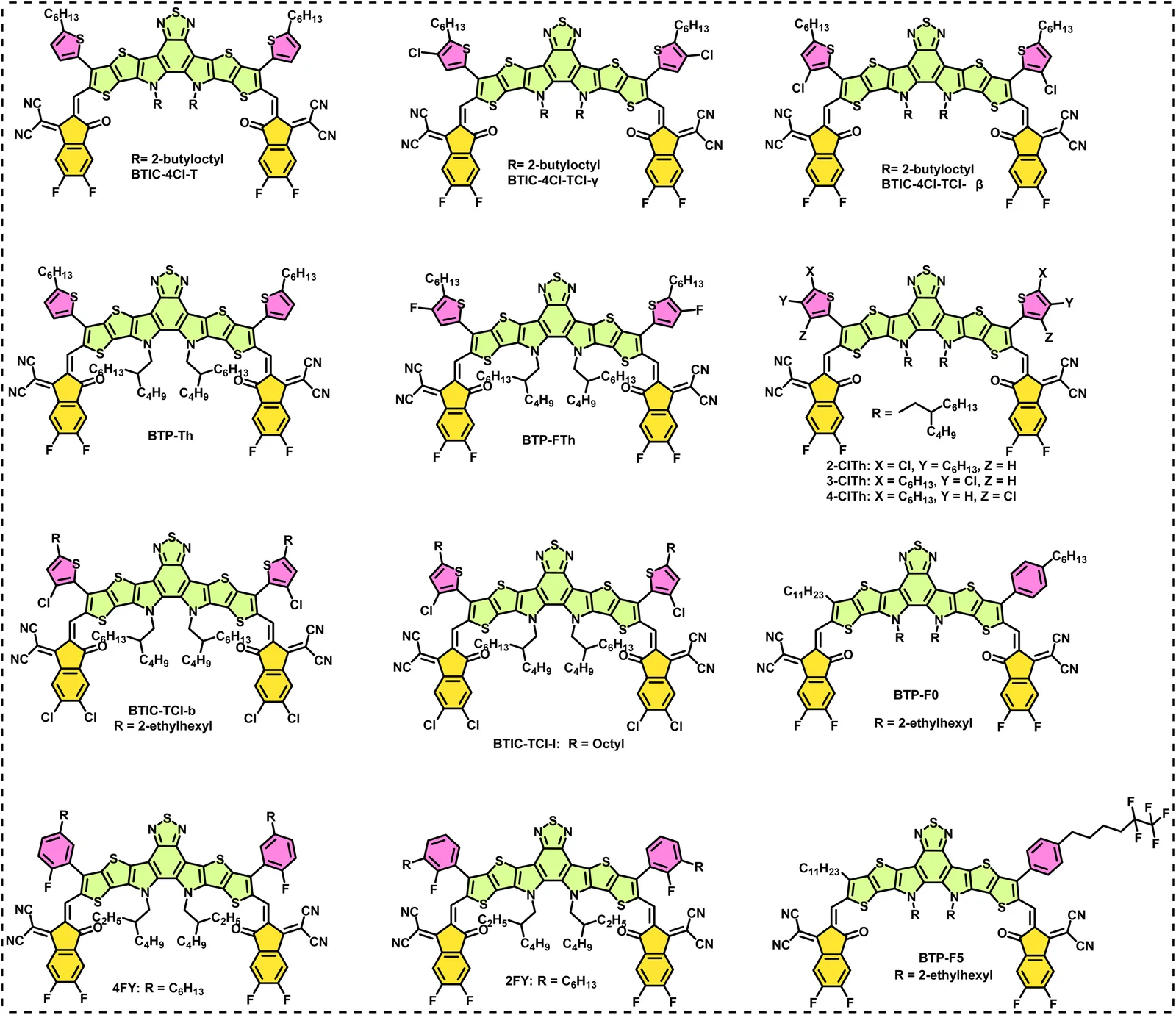
Chemical structures of side-chain halogenated NFREAs.

**Fig. 16:**
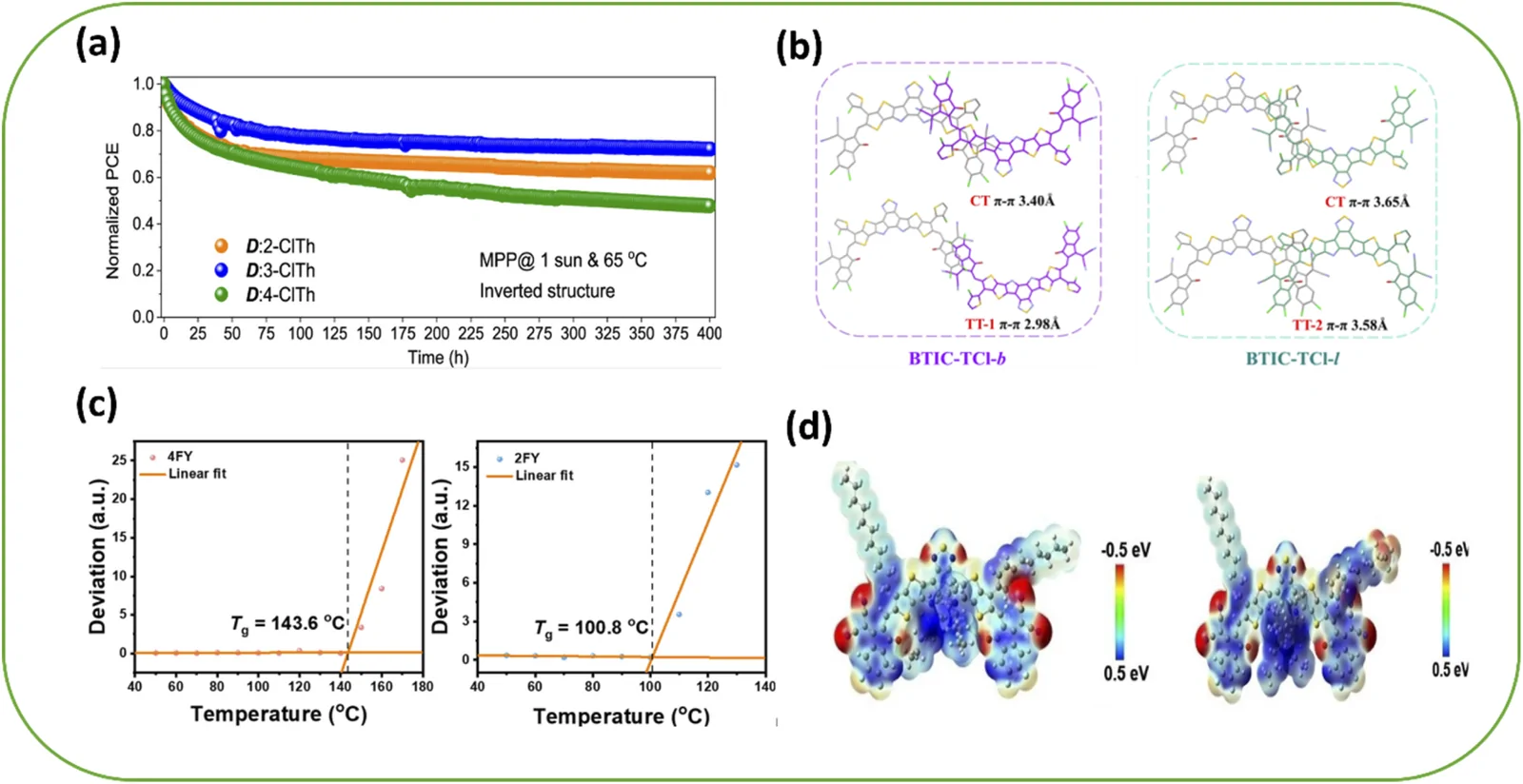
(a) MPP tracking of inverted devices measured under 1 sun illumination and 65 °C heating, reproduced with permission ref. 125, copyright 2023, American Chemical Society. (b) Distinct molecular packing modes of BTIC-TCl-b and BTIC-TCl-l revealed by single-crystal X-ray diffraction analysis, reproduced with permission ref. 126, copyright 2023, Royal Society of Chemistry; (c) glass transition temperatures (*T*_g_) of the 4FY and 2FY acceptors, reproduced with permission ref. 127, copyright 2023, American Chemical Society; (d) electrostatic potential maps of BTP-F0 and BTP-F5, reproduced with permission ref. 128, copyright 2024, Wiley-VCH GmbH.

### Phenazine-based Y-series NFAs for BHJ-OSCs

Phenazine-based fused cores have recently emerged as an attractive alternative to the benzothiadiazole-containing frameworks commonly used in Y-series acceptors. The larger fused aromatic structure of phenazine provides additional opportunities for extending conjugation and tuning intermolecular interactions, making it possible to simultaneously regulate molecular packing, charge transport, and low-energy-loss processes. As a result, phenazine-containing acceptors have attracted increasing attention as a promising platform for developing high-efficiency OSCs. One of the most effective approaches for optimizing these materials has been halogenation. While fluorination has long been employed to tune energy levels and intermolecular interactions, excessive fluorination can increase synthetic complexity and cost. Chlorine and bromine substitution offer attractive alternatives because of their larger atomic size and higher polarizability, which can strengthen intermolecular interactions and modify molecular packing more effectively. Although halogenation in Y-series acceptors is most commonly introduced at the terminal end groups, recent studies have shown that modifying the central phenazine-containing core can have an even more pronounced influence on molecular organization and device performance^129–134^.

For example, Chen *et al.* developed two structurally similar acceptors, CH4 and CH6, which differ only in the fluorination of the conjugation-extended phenazine core. The introduction of fluorine into the central unit not only lowered the frontier molecular orbital energy levels but also strengthened intermolecular interactions and increased the molecular dipole moment, as shown in Fig. 17a. As a result, CH6 exhibited enhanced crystallinity, more ordered molecular packing, and a well-developed fibrillar morphology in blend films. These improvements facilitated charge generation and transport while reducing energetic disorder, leading to a remarkable PCE of 18.33% for PM6:CH6-based devices compared with 16.49% for CH4. Notably, CH6 also maintained a high efficiency of 16.52% in 1 cm^2^ devices, highlighting the scalability of this molecular design strategy.^135^ The influence of the halogen species was further explored by Zou *et al.*, who developed CH-6F, CH-4Cl, and CH-6Cl. Although these acceptors share the same molecular backbone, replacing fluorine with chlorine led to distinctly different packing arrangements. Single-crystal analysis confirmed that chlorinated derivatives, CH-4Cl and CH-6Cl, exhibited a dual packing mode resulting in shorter intermolecular packing distances and more efficient charge-transport pathways than those observed for the fluorinated analogue, as shown in Fig. 17b and c. Consequently, the chlorinated acceptors exhibited more balanced charge-carrier transport, lower energetic disorder, and reduced non-radiative voltage losses. These advantages translated into outstanding device performance, and achieved a PCE of 17.72%, for CH-4Cl based devices it was further boosted with ternary devices, achieving a PCE of 18.22% and an exceptionally low non-radiative voltage loss of only 0.203 eV.^136^ where CH-6Cl and CH-6F based deviced achieved 17.22% and 16.77% PCE only. Further, Chen *et al.* expanded the molecular conjugation of the Y6 framework by extending both the central benzo[*c*][1,2,5]thiadiazole core and terminal units, yielding the A–D–A acceptor CH17. The enlarged π-conjugated architecture promoted a more compact three-dimensional molecular packing through a distinctive dual “end-unit-to-central-unit” interaction, as revealed by single-crystal analysis. This packing motif enhanced charge transport, reduced energetic disorder, and strengthened charge-transfer/local-exciton (CT/LE) state hybridization, thereby suppressing non-radiative recombination and lowering energy losses. As a result, PM6:CH17 devices achieved a PCE of 17.84%, outperforming the Y6 counterpart (16.27%), while ternary devices further reached 18.13% with a reduced energy loss of 0.49 eV and an enhanced *V*_OC_ of 0.889 V.^30^

**Fig. 17:**
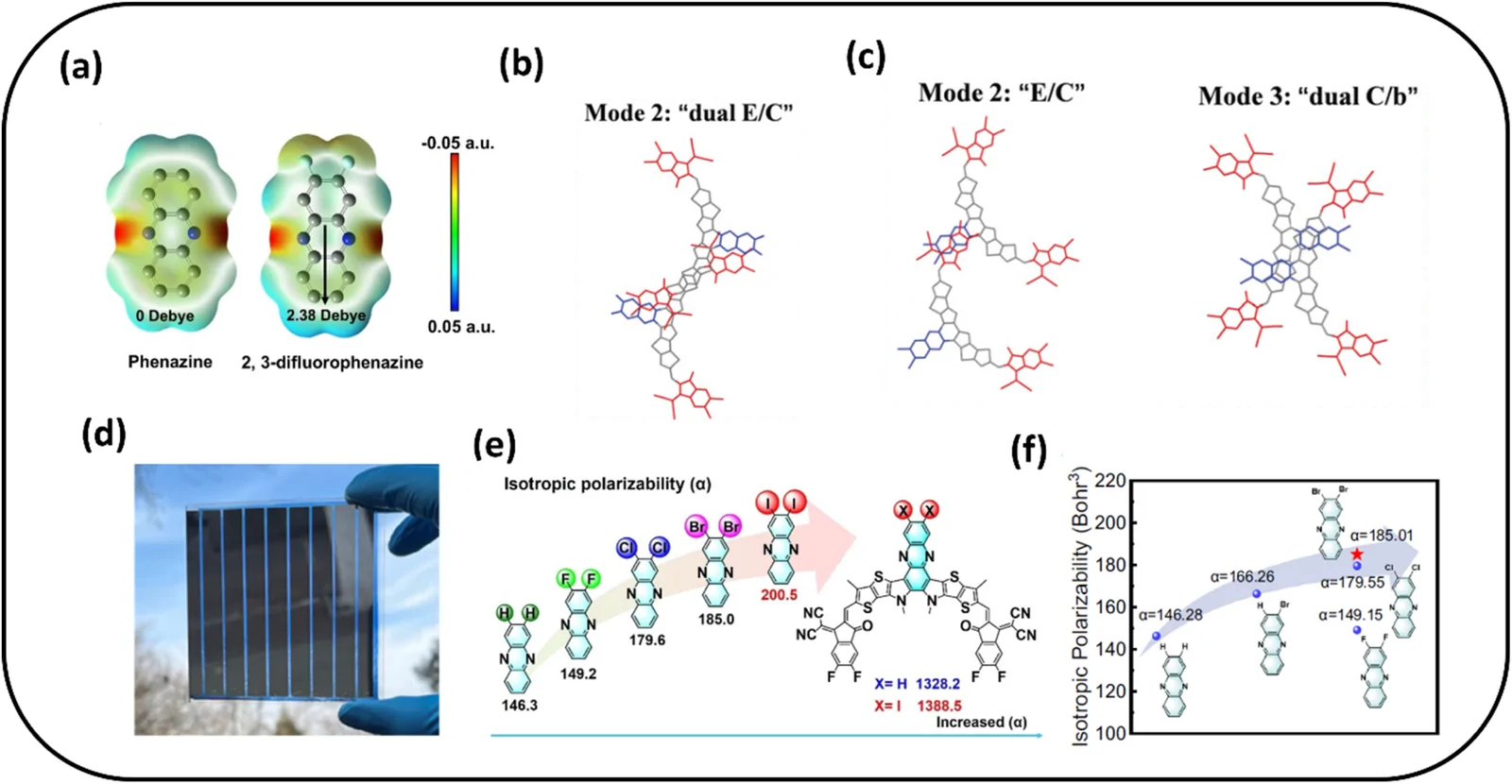
(a) ESP maps of the central units for CH4 and CH6, reproduced with permission, ref. 135, copyright 2022, Wiley-VCH GmbH; (b and c) distinct dual molecular packing motifs of CH-4Cl and CH-6Cl, revealed by single crystal X-ray diffraction analysis, reproduced with permission, ref. 136, copyright 2022, Royal Society Chemistry; (d) photograph of a large-area PM6:CH7 OSC module with an active area of 25.2 cm^2^, reproduced with permission ref. 137, copyright 2023 Wiley-VCH GmbH; (e) comparison of the isotropic polarizabilities of CH4 and CH45, including phenazine central units with different halogen substituents, reproduced with permission, ref. 140, copyright 2022, Royal Society Chemistry; (f) calculated isotropic polarizabilities of the acceptors, reproduced with permission ref. 46, copyright 2023, Springer Nature.

Besides core engineering, side-chain modification has also proven effective for further improving the performance and processability of phenazine-based Y-series acceptors. For example, Zhang *et al.* replaced the short alkyl substituent on the pyrrole ring of CH-6F with long branched alkyl chains to develop CH7 while retaining the conjugation-extended phenazine core. The introduction of bulky side chains significantly improved the solubility of CH7 in the environmentally friendly solvent *o*-xylene, which help to suppress the excessive molecular aggregation. As a result, CH7 formed a favorable bulk-heterojunction morphology with balanced charge transport, facilitating efficient charge generation and collection. Consequently, inverted PM6:CH7 devices processed from *o*-xylene achieved a PCE of 17.49%. More importantly, the excellent device performance could be successfully translated to large-area modules, with a 25.2 cm^2^ module delivering a PCE of 14.42% (Fig. 17d). CH7 also exhibited excellent operational stability, retaining 93% of its initial efficiency after 500 h of continuous illumination and 82% after thermal ageing at 65 °C for 500 h, highlighting the advantages of side-chain engineering for scalable and durable organic photovoltaics.^137^

In addition to improving processability, halogen engineering has been widely employed to further optimize intermolecular interactions and charge-transport properties within the CH-series acceptors. Building upon the high-performance CH14 platform, Liang *et al.* developed CH23 through a rational redistribution of halogen atoms between the central core and terminal groups. Although the molecular backbone remained largely unchanged, this subtle modification dramatically increased the molecular dipole moment from 0.12 D for CH14 to 2.48 D for CH23 and increased the packing density from 59.9% to 65.8%. The resulting increase in dielectric constant facilitated charge separation and promoted more balanced carrier transport. Consequently, CH23-based devices achieved an impressive PCE of 18.77% with a high fill factor of 80.45%, significantly outperforming the corresponding CH14 devices achieved a PCE of 17.81%, only.^138^

The importance of peripheral halogenation was further demonstrated by Yao *et al.* through the development of CH8F, in which all peripheral active sites of a two-dimensionally conjugation-extended CH-series framework were fluorinated. Compared with CH6F, CH8F exhibited substantially stronger intermolecular interactions and more ordered molecular packing, as evidenced by both single-crystal and GIWAXS analyses. The enhanced molecular ordering, together with an increased molecular dipole moment, resulted in higher packing density and a larger dielectric constant, reducing exciton binding energy and facilitating charge separation. Furthermore, CH8F-based blend films displayed shorter π–π stacking distances and larger crystal coherence lengths, indicative of more efficient charge-transport pathways and reduced recombination losses. As a result, D18:CH8F devices achieved a PCE of 18.80%, surpassing the corresponding D18:CH6F devices (17.91%). Owing to the excellent compatibility of CH8F with chlorinated donor polymers, incorporation of D18-Cl as a third component further enhanced molecular ordering and intermolecular π–π interactions, enabling ternary devices to reach a record PCE of 19.28% for CH-series acceptors.^139^ More recently, halogen engineering within phenazine-based acceptors has been extended beyond fluorine and chlorine. For instance, Chen and co-workers reported a iodinated acceptor CH45, which is the derivative of phenyl-fused YQx acceptor family. As shown in Fig. 17e, owing to the high polarizability of iodine, CH45 exhibited enhanced molecular polarizability and a larger dielectric constant than its non-iodinated analogue, resulting in reduced exciton binding energy and more efficient charge separation. These favorable characteristics facilitated charge generation and transport while suppressing recombination losses, enabling CH45-based devices to achieve a PCE of 18.15%, with the addition of 1-chloronaphthalene additive. Single-crystal and morphological analyses further revealed that the additive promotes tighter π–π stacking and improved crystalline ordering, thereby strengthening intermolecular interactions and contributing to the high FF observed in the corresponding devices.^140^

Beyond the identity of the halogen substituent, the fluorination pattern on the phenazine central unit has also been shown to strongly influence molecular packing and device performance. To explore this effect, Wang *et al.* developed a series of CH-based acceptors, CH-F, CH-CF, and CH-FCF, incorporating mono-fluorination, mono-trifluoromethylation, and combined fluorination/trifluoromethylation on the central phenazine unit, respectively. Although all three molecules share the same molecular framework, their packing behavior differs considerably. Among them, CH-FCF exhibited the highest degree of molecular ordering, with stronger π–π interactions, enhanced crystallinity, and a more pronounced fibrillar network morphology in PM6 blend films. These features facilitated charge generation and transport while suppressing energetic disorder. As a result, PM6:CH-FCF devices achieved a PCE of 18.41%, outperforming the corresponding CH-F (17.34%) and CH-CF (17.62%) devices.^141^ The influence of halogen substitution was further examined by Liang *et al.*, who introduced mixed-halogen patterns into the phenazine core through the design of CH-FC, CH-FB, and CH-CB, containing F/Cl, F/Br, and Cl/Br combinations, respectively. This approach was intended to take advantage of the different electronic and steric characteristics of each halogen atom. Single-crystal X-ray diffraction analysis revealed that CH-FB adopts the most compact molecular arrangement, exhibiting the shortest π–π stacking distance within the characteristic E/E packing motif. The tighter packing enhances intermolecular electronic coupling and facilitates multidirectional charge transport. Consistent with these structural observations, CH-FB-based blend films displayed more favorable nanoscale morphology, more efficient charge separation, and improved carrier transport than those based on CH-FC and CH-CB. Consequently, the PM6 devices incorporating CH-FB achieved a PCE of 18.26%, exceeding those obtained with CH-FC (18.10%) and CH-CB (17.93%). These results suggest that combining halogen atoms with complementary electronic and steric properties offers an effective route to fine-tune molecular packing without significantly altering the conjugated backbone.^142^ A similar trend was observed in the work of Liang *et al.*, who investigated the effect of stepwise bromination directly on the phenazine core through the development of CH20, CH21, and CH22. Unlike the more common strategy of brominating terminal groups, bromination at the central unit enhanced intermolecular interactions and dielectric properties while avoiding excessive aggregation. Increasing the degree of bromination progressively strengthened light absorption, lowered the frontier molecular orbital energy levels, and increased both molecular polarizability and the dielectric constant, as shown in Fig. 17f. As a result, dielectric screening became more effective, and exciton dissociation was facilitated. Among the three acceptors, CH22 exhibited the highest degree of molecular ordering, the shortest π–π stacking distance, and the lowest exciton binding energy, leading to more efficient charge generation and collection. Consequently, PM6:CH22 devices achieved a PCE of 19.06%, substantially higher than those based on CH20 (16.79%) and CH21 (18.12%). Notably, CH22 also displayed excellent thickness tolerance, maintaining a PCE of 15.70% even at an active-layer thickness of approximately 500 nm. These findings demonstrate that central-core bromination provides an effective means of simultaneously improving molecular packing, dielectric screening, and charge–transport properties, all of which are essential for high-performance and scalable OSCs.^46^

Beyond halogen engineering, extending the conjugation of quinoxaline- and phenazine-based cores has proven to be an effective approach for improving molecular packing and charge-transport properties. In this regard, Liu *et al.* expanded the quinoxaline-fused core of AQx-16 into a more π-extended phenazine framework, affording AQx-18 with a larger and more rigid conjugated backbone. Single-crystal and thin-film analyses revealed that the enlarged phenazine core promotes stronger intermolecular interactions and closer π–π contacts, leading to a more ordered three-dimensional charge-transport network. Importantly, these packing characteristics were largely retained in D18 blend films, which exhibited enhanced crystallinity, well-defined fibrillar nanostructures, and suitable phase separation. The resulting improvements in exciton dissociation, charge transport, and recombination suppression enabled D18:AQx-18 devices to achieve a PCE of 18.2%, with simultaneous increases in *V*_OC_, *J*_SC_, and FF. Further incorporation as a ternary component in the D18:L8-BO blend achieved an PCE of 19.1% and a high *V*_OC_ of 0.928 V.^143^ The importance of central-core halogenation was further demonstrated by Xu *et al.*, who developed a series of pyrido[2,3-*b*]quinoxaline (PyQx)-based acceptors, Py1–Py5, using a cascade chlorination strategy. Introducing chlorine atoms into the PyQx unit not only tuned the frontier molecular orbital energy levels but also altered the molecular dipole moment and intermolecular packing. Among the series, *ortho*-chlorinated Py2 exhibited the most favorable combination of molecular ordering and light-harvesting capability. Density functional theory calculations revealed that Py2 possesses a larger dipole moment and shorter intermolecular π–π stacking distance than the non-chlorinated and *meta*-/*para*-chlorinated analogues, leading to stronger intermolecular interactions and more compact packing. These features promoted efficient exciton dissociation, suppressed charge recombination, and yielded a PCE of 17.73%. Incorporation of Py2 into a ternary architecture further increased the efficiency to 18.43%, highlighting the effectiveness of central-core chlorination in regulating molecular organization and device performance.^144^ Xie *et al.* systematically investigated the impact of central-unit halogenation on the molecular packing and the photovoltaic performance of quinoxaline (Qx)-based NFAs. By varying the substitution positions of fluorine atoms on the central quinoxaline unit and modifying the terminal halogen groups, they developed a series of four NFAs, Qx-*o*-4F, Qx-*m*-4F, Qx-*p*-4F, and Qx-*p*-4Cl. The study revealed that the positional arrangement of fluorine atoms significantly influences the local dipole moment distribution, thereby regulating intermolecular interactions, molecular stacking modes, and optoelectronic properties. The results reveal that *para*-fluorinated central units promote stronger local dipole moments, thereby promoting more ordered molecular stacking compared with their *ortho*- and *meta*-substituted counterparts. Furthermore, replacing fluorinated end groups with chlorinated ones narrows the optical bandgap and reduces energy loss, facilitating a more favorable balance between *V*_OC_ and *J*_SC_. Among the series, Qx-*p*-4Cl exhibits red-shifted absorption, highly ordered molecular packing, and better blend morphology with polymer donor PM6, leading to efficient charge generation and transport. Consequently, PM6:Qx-*p*-4Cl devices achieve a PCE of 18.06%, with a *V*_OC_ of 0.879 V and *J*_SC_ of 25.63 mA cm^−2^, which is further enhanced to 18.78% through interface engineering.^145^ While the above studies focused primarily on molecular packing, Si *et al.* demonstrated that central-core engineering can also be used to suppress non-radiative energy losses. To this end, they developed CH-ThCl, an NFA containing a dichlorodithienoquinoxaline core with an extended and highly rigid π-conjugated framework. The combination of extended conjugation and chlorine substitution enhanced molecular rigidity and promoted J-aggregation, leading to improved photoluminescence efficiency and substantially reduced non-radiative recombination. As a result, PM6:CH-ThCl devices exhibited an exceptionally low non-radiative voltage loss of only 0.21 eV, enabling a high *V*_OC_ of 0.934 V and a PCE of 18.16%. To further optimize the morphology, CH-6F was introduced as a third component in PM6:CH6F blends, which enhances the miscibility and phase separation, facilitating more efficient charge generation and transport while preserving the high *V*_OC_. Consequently, the ternary devices achieved an enhanced PCE of 18.80%.^146^

Another effective approach for improving quinoxaline-based acceptors is the expansion of the fused π-conjugated core. Ding *et al.* introduced different aromatic building blocks into the quinoxaline framework to examine how conjugation extension influences molecular organization and device performance. Replacing the dibromophenanthrene unit in YB2B with a dibromobenzodithiophene moiety afforded YB2T, which possesses a larger and more planar conjugated framework. This structural modification broadened and red-shifted the absorption spectrum while narrowing the optical bandgap. More importantly, the extended aromatic framework strengthened intermolecular interactions and promoted tighter molecular packing. Single-crystal and thin-film studies revealed that YB2T forms a more ordered packing arrangement than YB2B, and these packing characteristics were largely preserved in PM6 blend films. The resulting active layers exhibited improved crystallinity and a more favorable phase-separated morphology, leading to more efficient charge extraction and reduced recombination. Consequently, PM6:YB2T devices achieved a PCE of 17.05%, with a *V*_OC_ of 0.918 V and a *J*_SC_ of 24.48 mA cm^−2^, substantially outperforming the corresponding YB2B devices (10.94%).^147^ Besides conjugation expansion, introducing halogenated thiophene units into the quinoxaline core provides another effective means of tuning molecular properties. Qiu *et al.* investigated this strategy through the development of Qx-H, Qx-Br, and Qx-Cl, in which thiophene, 2-bromothiophene, and 2-chlorothiophene units were incorporated into the central core, respectively. Relative to Qx-H, both halogenated derivatives exhibited broader absorption, deeper energy levels, and improved molecular ordering. These changes translated into more favorable blend morphologies and reduced energetic disorder, which helped suppress bimolecular recombination. Among the series, Qx-Br delivered the best overall balance between light harvesting, molecular packing, and charge transport, enabling PM6 devices to reach a PCE of 17.42% with a high *V*_OC_ of 0.915 V. Further incorporation of Y6 as a third component broadened the spectral response and improved photocurrent generation, resulting in a ternary-device efficiency of 18.36%.^148^ The importance of core fluorination was subsequently demonstrated by Liu and co-workers through a systematic study of the AQx-*n*F (*n* = 0–4) series, in which both the number and position of fluorine atoms on the quinoxaline core were precisely controlled. This work revealed that fluorination influences much more than energy-level alignment. Changes in fluorine content also affect molecular dipole moments, crystallinity, and the three-dimensional packing network, which govern charge-generation and recombination processes in blend films. Among the series, AQx-2F showed the favorable balance between crystallinity and donor–acceptor energetic offset. The enhanced dipole moment and well-developed three-dimensional packing network promoted the formation of a fibrous morphology with efficient exciton dissociation and charge transport, while limiting recombination losses. As a result, D18: AQx-2F devices achieved a PCE of 19.7%, which represented one of the highest efficiencies reported for a binary OSC.^149^ The molecular architecture and photovoltaic performance of the above-discussed acceptors as summarized in Table 5 and Fig. 18.

**Table 5:** Photovoltaic parameters of the acceptors discussed in this section

Donor	Acceptor	*J*_SC_ (mA cm^−2^)	*V*_OC_ (V)	FF (%)	PCE (%)	*E* ^opt^_g_ (eV)	HOMO (eV)	LUMO (eV)	*E*_loss_ (eV)	Ref.
PM6	CH4	26.11	0.88	71.1	16.49	1.40	−5.64	−3.83	0.51	135
	CH6	26.62	0.87	78.4	18.33	1.41	−5.68	−3.85	0.53	
PM6	CH-6F	25.31	0.87	75.99	16.77	1.41	−5.70	−3.85	0.557	136
	CH-4Cl	26.50	0.87	76.68	17.72	1.39	−5.74	−3.91	0.537	
	CH-6Cl	26.07	0.86	76.28	17.22	1.36	−5.79	−3.97	0.533	
PM6	CH17	26.19	0.88	77.2	17.84	∼1.35	−5.65	−3.86	0.49	30
	Y6	25.91	0.852	73.7	16.27	∼1.36	−5.68	−3.87	0.53	
PM6	CH7	24.95	0.881	76.88	17.49	∼1.35	−5.74	−3.75	—	137
PM6	CH14	26.35	0.869	77.79	17.81	1.37	−5.75	−3.85	0.538	138
	CH-23	26.64	0.876	80.45	18.77	1.39	−5.74	−3.82	0.537	
D18	CH-8F	26.01	0.899	80.38	18.80	∼1.36	−5.78	−3.80	—	139
	CH-6F	26.02	0.895	76.89	17.91	∼1.35	−5.74	−3.77	—	
PM6	CH-45	25.57	0.910	78.00	18.15	∼1.37	−5.66	−3.85	0.52	140
	CH-4	24.67	0.908	66.4	14.87	∼1.37	−5.64	−3.84		
PM6	CH-F	26.21	0.875	75.59	17.34	1.33	−5.62	−3.86	0.52	141
	CH-CF	26.07	0.886	76.30	17.62	1.37	−5.65	−3.85	0.53	
	CH-FCF	26.02	0.897	78.86	18.41	1.38	−5.67	−3.84	0.54	
PM6	CH-FC	26.31	0.886	77.66	18.10	—	−5.70	−3.85	—	142
	CH-FB	26.38	0.889	77.86	18.26	—	−5.69	−3.86	—	
	CH-CB	25.78	0.897	77.56	17.93	—	−5.67	−3.81	—	
PM6	CH-20	25.44	0.881	74.92	16.79	1.36	−5.61	−3.80	0.49	46
	CH-21	26.57	0.873	78.13	18.12	1.37	−5.63	−3.80	0.52	
	CH-22	26.74	0.884	80.62	19.06	1.37	−5.67	−3.83	0.53	
D18	AQx-18	25.00	0.938	77.80	18.20	∼1.40	−5.63	−3.85	0.537	143
	AQx-16	23.90	0.936	77.60	17.4	∼1.40	−5.61	−3.87	0.536	
PM1	Py1	25.29	0.736	63.3	11.78	1.33	−5.66	−4.04	—	144
	Py2	26.83	0.871	75.9	17.73	1.33	−5.73	−3.98	—	
	Py3	25.65	0.869	72.5	16.15	1.34	−5.73	−4.00	—	
	Py4	18.04	0.817	50.9	7.40	1.35	−5.71	−4.02	—	
	Py5	23.32	0.890	76.5	15.87	1.35	−5.78	−3.95	—	
PM6	Qx-*o*-4F	23.64	0.928	75.46	16.55	1.44	−5.73	−3.92	0.522	145
	Qx-*m*-4F	24.55	0.898	76.89	16.95	1.45	−5.69	−3.82	0.549	
	Qx-*p*-4F	24.89	0.877	79.46	17.34	1.47	−5.76	−3.91	0.560	
	Qx-*p*-4Cl	25.63	0.879	80.17	18.06	1.41	−5.65	−3.96	0.535	
PM6	CH-ThCl	25.43	0.934	76.45	18.16	∼1.38	−5.67	−3.78	0.519	146
PM6	YB2B	19.30	0.953	59.46	10.94	1.43	−5.75	−3.86	0.602	147
	YB2T	24.48	0.918	75.85	17.05	1.38	−5.73	−3.89	0.552	
PM6	Qx-H	20.78	0.922	73.77	14.11	—	−5.59	−3.92	0.521	148
	Qx-Br	24.27	0.915	78.47	17.42	—	−5.64	−3.95	0.526	
	Qx-Cl	24.13	0.913	78.32	17.25	—	−5.65	−3.95	0.533	
PM6	AQx-0F	23.2	0.961	73.5	16.4	—	−5.57	−3.84	0.482	149
	AQx-1F	24.3	0.947	76.9	17.7	—	−5.60	−3.86	0.506	
	AQx-2F	26.1	0.937	80.4	19.7	—	−5.62	−3.88	0.518	
	AQx-3F	23.3	0.946	75.0	16.5	—	−5.65	−3.90	0.539	
	AQx-4F	17.6	0.991	63.1	11.0	—	−5.72	−3.94	0.532	

**Fig. 18:**
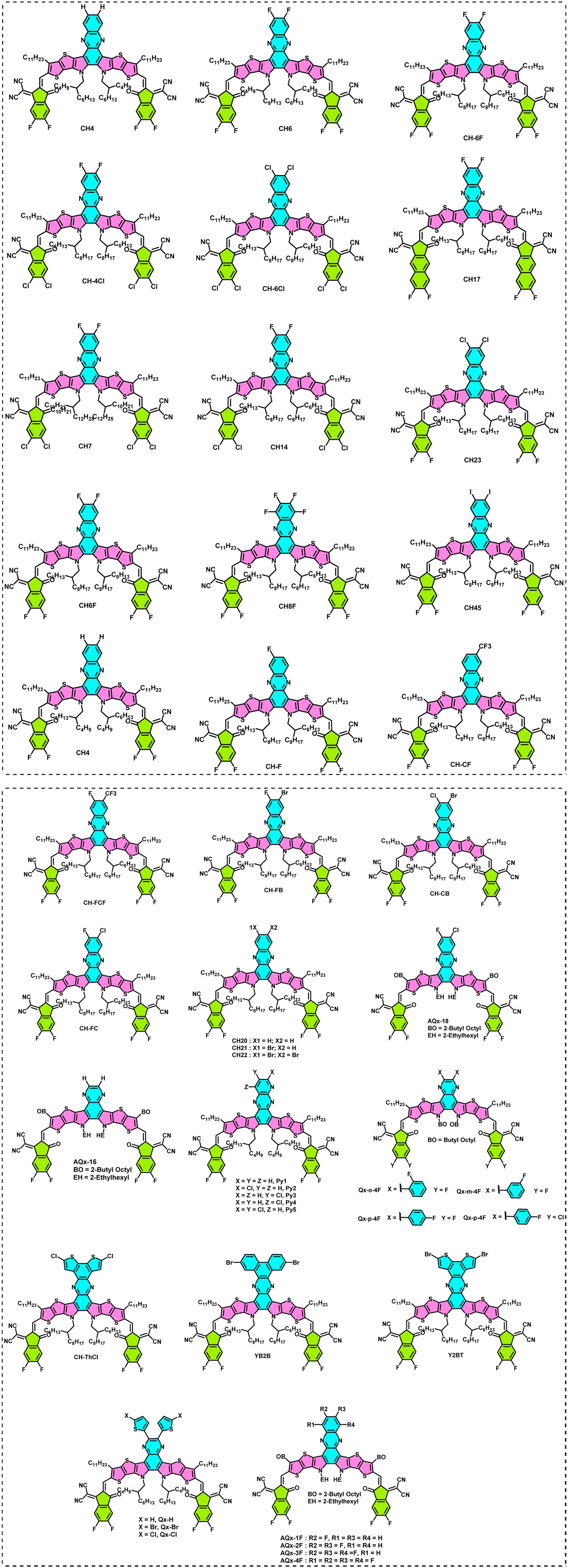
Chemical structures of halogen-incorporated acceptor molecules.

## Conclusions and future perspective

The introduction of NFAs has marked a golden era in the development of OSCs. When combined with polymer/small molecular donors, the performance of OSCs has reached up to 20%. However, despite their outstanding performance, numerous challenges remain, such as multistep synthesis, low-product yields, complicated processing methods, poor long-term stability, and limited performance of large-area devices, which collectively affect the future commercialization of OSCs.

According to their molecular architectures, NFAs are classified into two types: fused-ring acceptors and non-fused-ring acceptors. Compared to non-fused-ring acceptors, the fused-ring acceptors gained significant attention due to their planar molecular structure and high photovoltaic performance. The well-known and efficient fused-ring NFAs are ITIC and Y6 molecules. These fused-ring NFAs typically have a highly rigid and more planar central backbone unit, electron-donating groups that are attached perpendicular to the central backbone unit, and strong electron-withdrawing groups flanked on both sides.

To further improve the performance and stability of OSCs, the future optimization of NFAs should be focused on the design of novel central building blocks, end group engineering and side chain engineering.

(i) The planar fused central core is useful for achieving high electron mobility through efficient intramolecular charge transport, particularly π–π interaction, and molecular packing. However, an excessively large core size can induce long-range ordering, strong aggregation, and over-large packing, which hinder exciton dissociation and lead to a low FF. To address this issue, strategies such as appropriate core size design, heteroatom introduction, and rational isomerization are the best methods. In addition, simple and green synthesis of the central core is urgently needed to be explored.

(ii) The terminal electron-withdrawing groups facilitate efficient intermolecular charge transport *via* strong molecule contacts. Therefore, the incorporation of halogens (F and Cl) into the terminal acceptor unit is an important method to enhance absorption and improve molecular packing, thereby increasing charge mobility.^150^ In addition, extending end-groups through aromatic ring fusion can expand the conjugated system and moderately enhance intermolecular interactions.

(iii) The side chain substituents attached to the core unit are important for improving molecular solubility and device performance by tuning the molecular aggregation and phase separation. Compared with aromatic side chains, alkoxy side chains show weaker intermolecular interactions, which can facilitate improved conjugation and aggregation.

Therefore, as summarized above, the design of novel NFA molecules, such as ITIC and Y6, can be regarded as an effective method to promote the development of OSCs. Even though these state-of-the-art NFAs show a visible-near-infrared absorption range, high molar absorption coefficients, and excellent device performances, they still suffer from tedious synthetic routes, high cost, and critical device fabrication, which significantly limit their commercial viability. Therefore, future NFAs should be designed to be more synthetically accessible, potentially through the use of modern organic transformations such as C-H activation and photoredox synthesis. A key challenge lies in the rational design of simple core structures that simultaneously enable sufficient light harvesting, appropriate aggregation behavior, and favorable film morphology. The band gap of the NFA should be precisely tuned through appropriate D and A units, then matched with the polymer donor. While the *J*_SC_ and FF values of OSCs are approaching those of high-efficiency inorganic and perovskite solar cells, further reduction of *V*_OC_ loss remains essential. This can be achieved by choosing appropriate D and A molecules in OSC device construction. In addition, the potential NFA requires good processability in green solvents and film thickness fluctuation during large-area printing. Moreover, the device stability of efficient polymer: NFA blends needs to be further improved under illumination and thermal stress, which may be realized in the new type of NFA.

## Conflicts of interest

The authors declare no conflict of interest.

## Data Availability

No primary research results, software or code have been included and no new data were generated or analysed as part of this review. All information discussed in this article is available from the cited references.
